# Leaf cDNA-AFLP analysis of two citrus species differing in manganese tolerance in response to long-term manganese-toxicity

**DOI:** 10.1186/1471-2164-14-621

**Published:** 2013-09-14

**Authors:** Chen-Ping Zhou, Yi-Ping Qi, Xiang You, Lin-Tong Yang, Peng Guo, Xin Ye, Xin-Xing Zhou, Feng-Jiao Ke, Li-Song Chen

**Affiliations:** 1 Institute of Horticultural Plant Physiology, Biochemistry and Molecular Biology, Fujian Agriculture and Forestry University, 350002 Fuzhou, China; 2 College of Horticulture, Fujian Agriculture and Forestry University, 350002 Fuzhou,, China; 3 Institute of Materia Medica, Fujian Academy of Medical Sciences, 350001 Fuzhou, China; 4 College of Life Science, Fujian Agriculture and Forestry University, 350002 Fuzhou, China; 5 College of Resources and Environmental Sciences, Fujian Agriculture and Forestry University, 350002 Fuzhou, China

**Keywords:** cDNA-AFLP, *Citrus grandis*, *Citrus sinensis*, Leaves, Manganese

## Abstract

**Background:**

Very little is known about manganese (Mn)-toxicity-responsive genes in citrus plants. Seedlings of ‘Xuegan’ (*Citrus sinensis*) and ‘Sour pummelo’ (*Citrus grandis*) were irrigated for 17 weeks with nutrient solution containing 2 μM (control) or 600 μM (Mn-toxicity) MnSO_4_. The objectives of this study were to understand the mechanisms of citrus Mn-tolerance and to identify differentially expressed genes, which might be involved in Mn-tolerance.

**Results:**

Under Mn-toxicity, the majority of Mn in seedlings was retained in the roots; *C. sinensis* seedlings accumulated more Mn in roots and less Mn in shoots (leaves) than *C. grandis* ones and Mn concentration was lower in Mn-toxicity *C. sinensis* leaves compared to Mn-toxicity *C. grandis* ones. Mn-toxicity affected *C. grandis* seedling growth, leaf CO_2_ assimilation, total soluble concentration, phosphorus (P) and magenisum (Mg) more than *C. sinensis*. Using cDNA-AFLP, we isolated 42 up-regulated and 80 down-regulated genes in Mn-toxicity *C. grandis* leaves. They were grouped into the following functional categories: biological regulation and signal transduction, carbohydrate and energy metabolism, nucleic acid metabolism, protein metabolism, lipid metabolism, cell wall metabolism, stress responses and cell transport. However, only 7 up-regulated and 8 down-regulated genes were identified in Mn-toxicity *C. sinensis* ones. The responses of *C. grandis* leaves to Mn-toxicity might include following several aspects: (1) accelerating leaf senescence; (2) activating the metabolic pathway related to ATPase synthesis and reducing power production; (3) decreasing cell transport; (4) inhibiting protein and nucleic acid metabolisms; (5) impairing the formation of cell wall; and (6) triggering multiple signal transduction pathways. We also identified many new Mn-toxicity-responsive genes involved in biological and signal transduction, carbohydrate and protein metabolisms, stress responses and cell transport.

**Conclusions:**

Our results demonstrated that *C. sinensis* was more tolerant to Mn-toxicity than *C. grandis*, and that Mn-toxicity affected gene expression far less in *C. sinensis* leaves. This might be associated with more Mn accumulation in roots and less Mn accumulation in leaves of Mn-toxicity *C. sinensis* seedlings than those of *C. grandis* seedlings. Our findings increase our understanding of the molecular mechanisms involved in the responses of plants to Mn-toxicity.

## Background

Manganese (Mn), which is the twelfth abundant element and the third most common element in the Earth’s crust, is absorbed mainly as Mn^2+^ by plant roots [1]. Mn, an essential trace element for the normal growth and development of higher plant, is involved in many biochemical processes. With decreasing pH, the amount of exchangeable Mn (mainly Mn^2+^ form) increases in the soil solution [2]. Like other heavy metals, however, Mn is harmful to most of the plants when present in excess [3,4]. After aluminum (Al), Mn-toxicity is probably the most important factor limiting plant productivity in acidic soils, which comprise up to 50% of the world’s potentially arable lands [5]. Furthermore, the acidity of the soils is gradually increasing due to rapid industrialization, the emission of acidic gases and consequently acid deposition [6].

Disturbance of plant metabolism by Mn-toxicity happens in multiple ways. Toxic effects of Mn on plants include inhibition of growth, transpiration and photosynthesis [4,7-9], apoplastic deposition of oxidized Mn and phenolics [10,11], induction of oxidative stress through direct generation of reactive oxygen species (ROS) [3,12-16], interfering with the absorption, translocation, and use of other mineral elements [4,15,17,18], impairment of leaf structure and chloroplast ultrastructure [18,19], alteration of hormone balances [18,20,21], modification of enzyme (i.e. ribulose-1,5-bisphosphate carboxylase/oxygenase [7,8], Mn-dependent superoxide dismutase [22], oxalate oxidase [23], indole-acetic acid oxidase [20], glycosy transferase [24], IAA-amino acid hydrolases [25], phenylalanine ammonia-lyase [26], phospho*enol*pyruvate carboxykinase [27] and Mn-peroxidase [10]) activities, and affecting carbohydrate, amino acid, protein and nucleic acid metabolisms [4,8,10,13,14,17,18,28].

To deal with heavy metal stresses, plants have evolved a considerable degree of developmental plasticity, including adaptive responses *via* cascades of molecular networks [29]. Increasing evidence shows that plant responses to heavy metal stresses is associated with changes in the expression profiles of genes involved in a broad spectrum of physiological, biochemical and cellular processes including carbohydrate and energy metabolism, photosynthesis, protein biosynthesis and degradation, nucleic acid metabolism, signal transduction, transcriptional regulation, cell transport and stress responses [30,31]. However, limited data are available on the differential expression of genes in response to Mn-toxicity in plants.

Techniques for gene expression analyses in plants have been widely explored. cDNA-amplified fragment length polymorphism (cDNA-AFLP), which does not require prior sequence information, is an efficient, sensitive, and reproducible technology for the discovery and identification of genes based on their polymorphism or differential expression patterns [32]. This technique is a robust and high-throughput tool for analysis of genome-wide gene expression fluctuation induced by a specific stress and is also a useful tool for the isolation of novel genes [30,31,33].

Citrus belongs to evergreen subtropical fruit trees and is cultivated in humid and subhumid of tropical, subtropical, and temperate regions of the world mainly on acidic soils. Although the effects of Mn-toxicity on citrus chloroplast ultrastructure, CO_2_ assimilation, carbohydrates, photosynthetic electron transport and antioxidant systems have been investigated [8,19], very little is known about Mn-toxicity-responsive genes in citrus plants. In this study, we investigated the effects of Mn-toxicity on growth, leaf CO_2_ assimilation, leaf concentrations of malondialdehyde (MDA), chlorophyll (Chl) and total soluble protein, root, stem and leaf concentration of Mn, leaf phosphorus (P) and magnesium (Mg) concentrations, and expression of leaf genes revealed by cDNA-AFLP in *Citrus grandis* and *Citrus sinensis* seedlings having different Mn-tolerance. The objectives of this study were to understand the mechanisms of citrus Mn-tolerance and to identify differentially expressed genes, which might be involved in Mn-tolerance.

## Results

### Plant growth, root, stem and leaf Mn concentration, and leaf Mg and P concentrations

**Figure 1:**
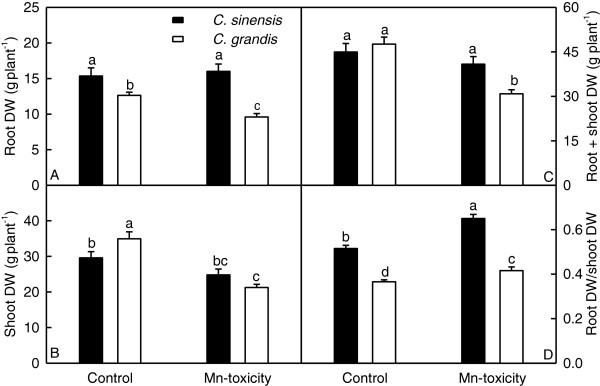
**Effects of Mn**-**toxicity on growth of *Citrus grandis *and *C. sinensis *seedlings. (A-C)** Root, shoot and root + shoot DW. **(D)** Ratio of root DW to shoot DW. Bars represent means ± SE (*n* = 10). Different letters above the bars indicate a significant difference at *P* < 0.05.

For *C. grandis*, Mn-toxicity decreased root, shoot and whole plant (root + shoot) dry weight (DW), and increased the ratio of root DW to shoot DW. However, Mn-toxicity did not significantly affect root, shoot and whole plant DW in *C. sinensis* seedlings except for increased ratio of root DW to shoot DW. Root DW, whole plant DW and the ratio of root DW to shoot DW were higher in *C. sinensis* seedlings than in *C. grandis* ones or similar between two species, except that shoot DW was lower in the former at the 2 μM Mn treatment (Figure 1). In addition, a few *C. grandis* leaves from the minority of Mn-toxicity plants became interveinal chlorosis or necrotic blotching of foliage, while no visible symptoms occurred in Mn-toxicity *C. sinensis* leaves (Additional file 1).

**Figure 2:**
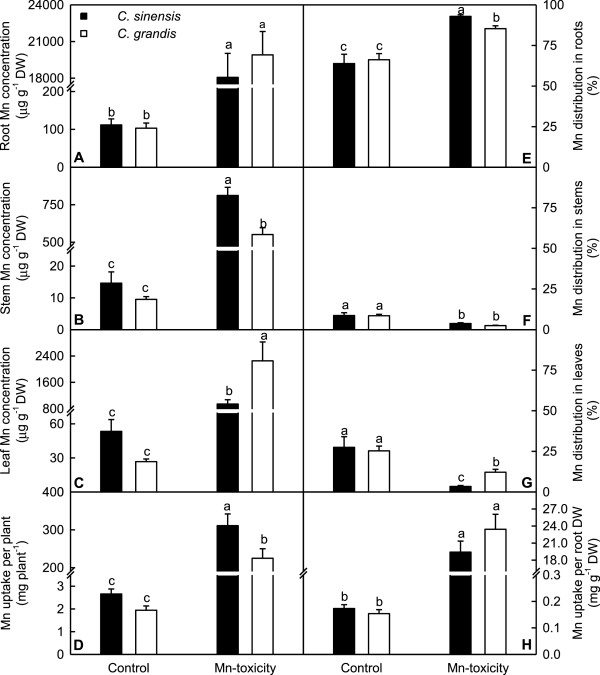
**Effects of Mn**-**toxicity on root, stem and leaf Mn concentration, Mn uptake and Mn distribution. (A**-**C)** Root, stem and leaf Mn concentration. **(D)** Mn uptake per plant. **(E**-**G)** Mn distribution in roots, stems and leaves. **(H)** Mn uptake per root DW. Bars represent means ± SE (*n* = 4). Different letters above the bars indicate a significant difference at *P* < 0.05.

As shown in Figure 2, Mn-toxicity increased root, stem and leaf Mn concentration, Mn distribution in roots, Mn uptake per plant and per root DW, and decreased Mn distribution in stems and leaves. Under control condition, all these parameters did not significantly differ between *C. grandis* and *C. sinensis* seedlings. When exposed to Mn-toxicity, stem Mn concentration, Mn uptake per plant and Mn distribution in roots were higher in *C. sinensis* seedlings than in C. *grandis* ones, while leaf Mn concentration and Mn distribution in leaves were lower in the former than in the latter.

**Figure 3:**
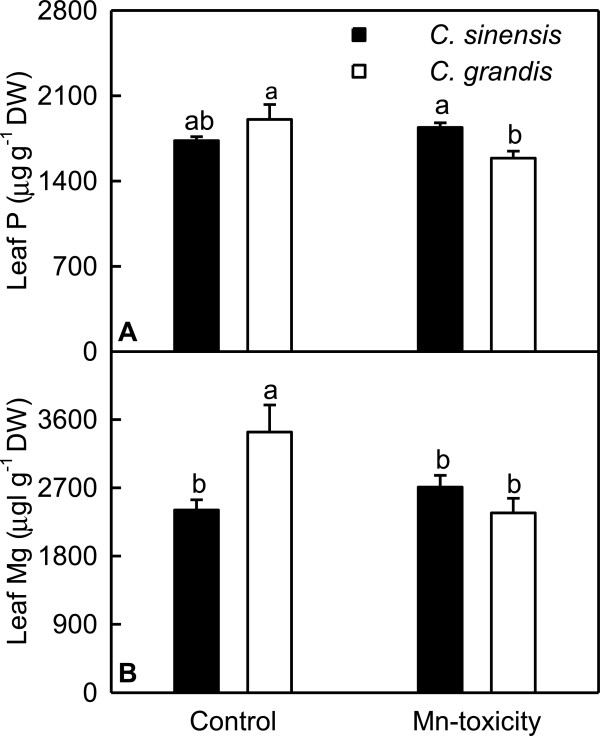
**Effects of Mn-toxicity on P (A) and Mg (B) concentrations in leaves.** Bars represent means ± SE (*n* = 4). Different letters above the bars indicate a significant difference at *P* < 0.05.

Mn-toxicity decreased P and Mg concentrations in *C. grandis* leaves, but did not significantly affect them in *C. sinensis* leaves (Figure 3).

### Leaf total soluble protein, MDA and Chl concentrations and gas exchange

**Figure 4:**
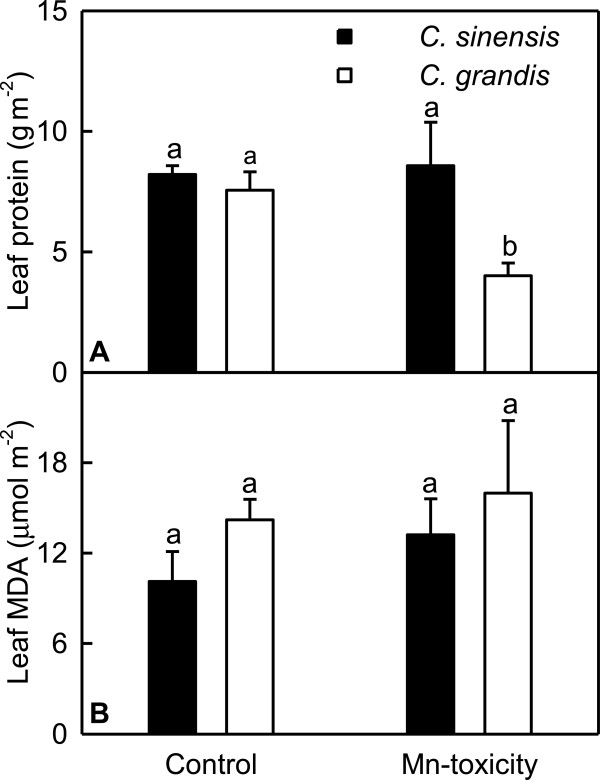
**Effects of Mn-toxicity on total soluble protein (A) and MDA (B) concentrations in leaves.** Bars represent means ± SE (*n* = 4). Different letters above the bars indicate a significant difference at *P* < 0.05.

Total soluble protein concentration was decreased by Mn-toxicity in *C. grandis* leaves, but was not significantly affected in *C. sinensis* ones (Figure 4A). As shown in Figure 4B, Mn-toxicity did not significantly affect leaf concentration of MDA.

**Figure 5:**
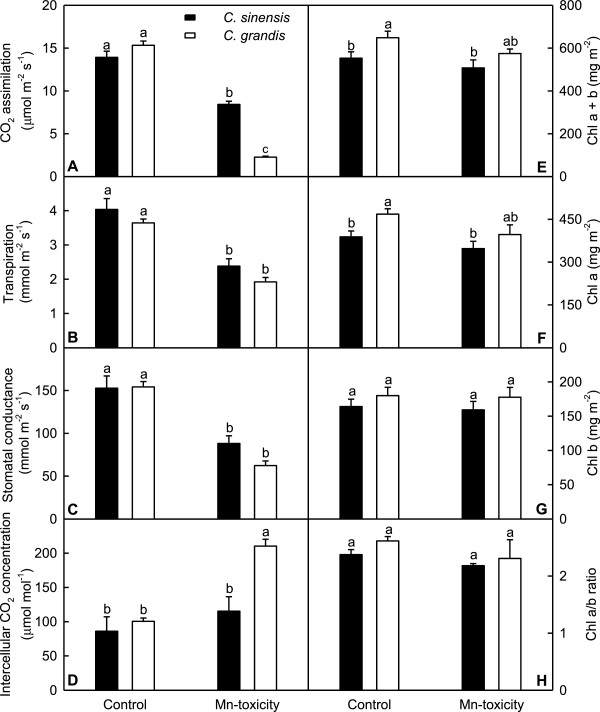
**Effects of Mn**-**toxicity on gas exchange and Chl of *Citrus grandis *and *C. sinensis *leaves. (A**-**D)** Leaf CO_2_ assimilation, transpiration, stomatal conductance and intercellular CO_2_ concentration. **(E**-**G)** Leaf concentrations of Chl a + b, Chl a and Chl b. **(H)** Leaf ratio of Chl a/b. Bars represent means ± SE (*n* = 4 or 5 ). Different letters above the bars indicate a significant difference at *P* < 0.05.

Mn-toxicity decreased CO_2_ assimilation (Figure 5A), transpiration (Figure 5B) and stomatal conductance (Figure 5C) in leaves of *C. grandis* and *C. sinensis*, especially in the former. Mn-toxicity increased intercellular CO_2_ concentration in *C. grandis* leaves, but did not significantly affect it in *C. sinensis* leaves (Figure 5D). Mn-toxicity did not significantly affect leaf concentrations of Chl a+b, Chl a and Chl b, and the ratio of Chl a to Chl b in the two citrus species (Figure 5E-H).

### Identification of differentially expressed genes by cDNA-AFLP

**Figure 6:**
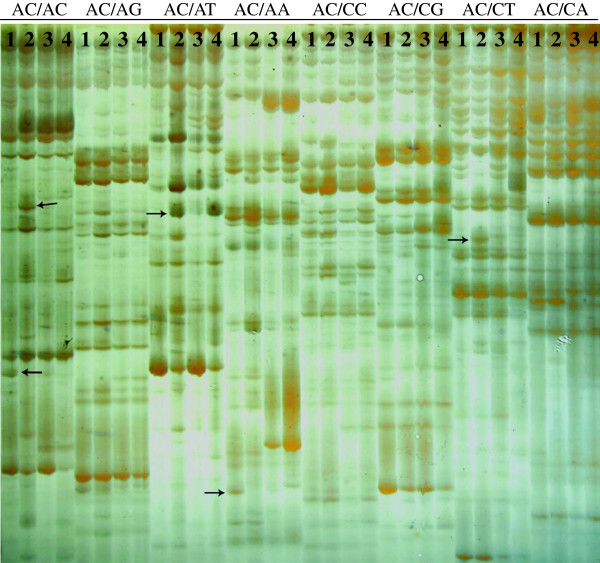
**cDNA**-**AFLP profiles using one *Eco*R I selective primer and eight *Mes *I selective primers.** One *Eco*R I selective primer: *Eco*R I-AC; Eight *Mes* I selective primers: *Mes* I-AC, AG, AT, AA, CC, CG, CT and CA; 1: Control leaves of *Citrus grandis*; 2: Mn-toxicity leaves of *C. grandis*; 3: Control leaves of *C. sinensis*; 4: Mn-toxicity leaves of *C. sinenis*; Arrows indicate differentially expressed TDFs.

A total of 256 selective primer combinations were used for the cDNA-AFLP analysis in order to identify the genes responsive to Mn-toxicity in the leaves of two citrus species differing in Mn-tolerance (Figure 6). For *C. grandis* leaves, a total of 16–37 (an average of 21.4) clear and unambiguous transcript-derived fragments (TDFs) were detected with each premier combination, which resulted in approximately 5490 TDFs, ranging from 100–750 bp. A total of 223 differentially expressed and reproducible TDFs were recovered from the silver-stained cDNA-AFLP gels based on their presence, absence or difference in the levels of expression. All these TDFs were re-amplified, ligated and sequenced, and 213 cDNA fragments produced useable sequence data. TDF sequences were compared with those present in the GenBank database (Additional file 2). Of the 213 TDF sequences, 99 TDFs showed significant homology to genes encoding known or putative proteins, and 23 TDFs were homologous to genes encoding uncharacterized proteins, hypothetical proteins or unknown proteins. The remaining 91 TDFs did not show homology to any nucleotide or amino sequence in the public databases. Of these 122 matched TDFs, 42 (34.4%) TDFs increased and 80 (65.6%) decreased in response to Mn-toxicity. According to the biological functional properties, these TDFs were classified into the following functional categories: biological regulation and signal transduction (18.9%), carbohydrate and energy metabolism (10.7%), nucleic acid metabolism (4.1%), protein metabolism (17.2%), lipid metabolism (0.8%), cell wall metabolism (8.2%), stress responses (10.7%), cell transport (6.6%), other and unknown biological processes (23.0%) (Additional file 2, Figure 7B).

**Figure 7:**
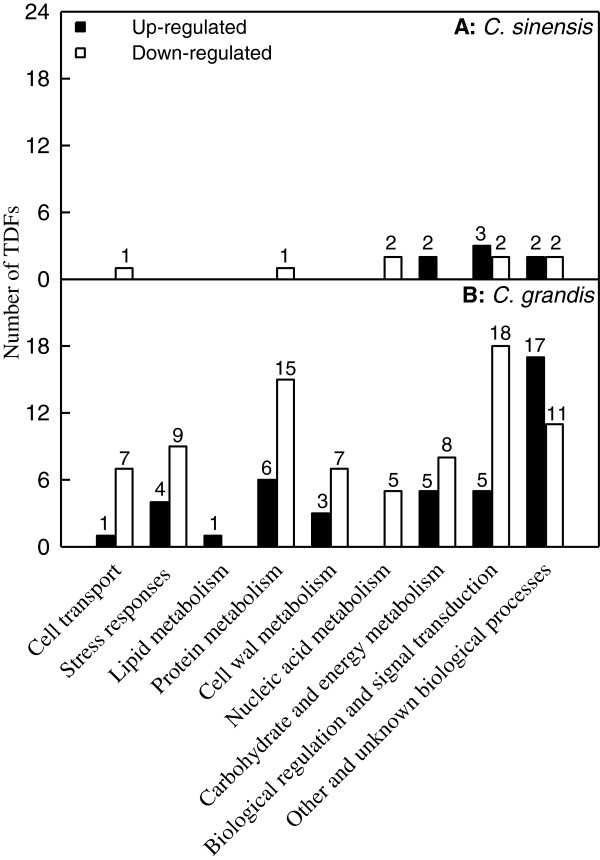
**Differentially expressed genes in Mn**-**toxicity citrus leaves as compared with control ones.**

For *C. sinensis* leaves, a total of 16–37 (an average of 21.3) clear and unambiguous TDFs were obtained with each premier pair, which yield approximately 5450 TDFs, ranging from 100–750 bp. A total of 20 differentially expressed and reproducible TDFs were isolated from the silver-stained cDNA-AFLP gels. All these TDFs were sequenced, and produced readable sequences (Additional file 3). Of the 20 TDFs, 12 TDFs were homologous to genes encoding known or putative proteins, and three TDFs belonged to genes encoding hypothetical proteins. The remaining five TDFs had no database matches. Among the 15 matched TDFs, seven (46.7%) TDFs were up-regulated and eight (53.3%) was down-regulated by Mn-toxicity. These TDFs were involved in biological regulation and signal transduction (33.3%), carbohydrate and energy metabolism (13.3%), nucleic acid metabolism (13.3%), protein metabolism (6.7%), cell transport (6.7%), other and unknown biological processes (26.7%) (Additional file 3, Figure 7A).

### qRT-PCR analysis of some Mn-toxicity-responsive genes

**Figure 8:**
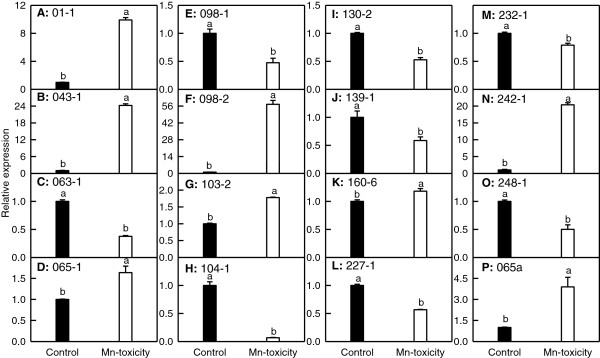
**Relative expression of TDFs from *Citrus grandis *(A**-**O) and from *C. sinensis *(P) leaves.** Relative expression of genes encoding ATP synthase subunit alpha (TDF #01-1; **A**), glycoside hydrolase family 28 protein (TDF #043-1; **B**), anthranilate phosphoribosyltransferase-like protein (TDF #063-1; **C**), ATP synthase subunit alpha (Atp 1; TDF #065-1; **D**), monodehydroascorbate reductase (TDF #098-1; **E**), xylem cysteine proteinase 2 (TDF #098-2; **F**), catalase (TDF #103-2; **G**), peroxidase 42 (TDF #104-1; **H**), NADP-dependent alkenal double bond reductase P2 (TDF #130-2; **I**), DNA polymerase phi subunit (TDF #139-1; **J**), regulator of Vps4 activity in the MVB pathway protein (TDF #160-6; **K**), glutathione S-transferase Tau2 (TDF #227-1; **L**), Myb family transcription factor (TDF #232-1; **M**), cell wall-associated hydrolase (TDF #242-1; **N**), ABC transporter family protein (TDF #248-1; **O**) in *C. grandis* leaves and Atp1 (TDF #065a; **P**) in *C. sinensis* leaves. Bars represent means ± SE (*n* = 3). Different letters above the bars indicate a significant difference at *P* < 0.05.

To validate the cDNA-AFLP expression patterns, 15 TDFs from *C. grandis* leaves and one TDF from *C. sinensis* ones were selected for qRT-PCR analysis. The expression levels of all these TDFs except for two TDFs (TDFs # 232–1 and 160–6) matched well with the expression profiles observed with cDNA-AFLP (Figure 8). The discrepancy in expression patterns for two TDFs between qRT-PCR and cDNA-AFLP analysis might be due to gene family complexity. Nevertheless, the cDNA-AFLP technique allowed us to isolate the differentially expressed genes under Mn-toxicity.

## Discussion

### *C. sinensis* is more tolerant to Mn-toxicity than *C. grandis*

As shown in Figures 1 and 2A-C, 600 μM Mn treatment greatly inhibited *C. grandis* plant growth, especially the shoots and increased the concentration of Mn in roots, stems and leaves, and foliar Mn concentration for 600 μM Mn treatment was far more than the sufficiency range of 25–200 mg kg^-1^ DW for sweet orange (*C. sinensis*) leaves [34]. Based on these results, plants that received 600 μM Mn are considered Mn-excess (Mn-toxicity). Mn-toxicity-induced decrease in plant DW and increase in root DW/shoot DW ratio (Figure 1) agree with the previous results obtained on *C. grandis*[8] and with the view that Mn-toxicity affects plant tops more than root systems [4]. Mn-toxicity, however, had no influence on the ratio of root DW to shoot DW in lucerne (*Medicago sativa*) plants, despite Mn-toxicity depressed growth of shoots and roots [35].

Our results showed that Mn-toxicity *C. grandis* plants had decreased root, shoot and root + shoot DW, and increased ratio of root DW to shoot DW, while Mn-toxicity did not significantly affect *C. sinensis* growth except for increased root DW/shoot DW ratio (Figure 1), meaning that *C. sinensis* is more tolerant to Mn-toxicity than *C. grandis*. This is also supported by our data that the gas exchange in *C. grandis* leaves was affected by Mn-toxicity far more than in *C. sinensis* ones (Figure 5A-D), and that Mn-toxicity decreased total soluble protein, P and Mg concentrations only in *C. grandis* leaves (Figures 3 and 4A). Like that of previous workers [8,36,37], the observed lower CO_2_ assimilation in Mn-toxicity leaves of *C. grandis* and *C. sinensi*s was primarily caused by non-stomatal factors because the decreases in both CO_2_ assimilation and stomatal conductance was accompanied by unchanged or increased intercellular CO_2_ assimilation (Figure 5A, C and D). It is noteworthy that the reduction in CO_2_ assimilation in leaves of Mn-toxicity plants could not attributed to photo-oxidative damage and decreased Chl, because there were no significant differences in leaf concentrations of MDA (a marker of peroxidative damage), Chl a+b, Chl a and Chl b (Figures 4B and 5E-G) between control and Mn-toxicity leaves. Similar results have been obtained on *C. grandis*[8].

Under Mn-toxicity, the majority of Mn in *C. sinensis* and *C. grandis* plants was retained in the roots (Figure 2E), as previously found for *C. grandis*[8], lucerne [35] and Douglas fir [38]. However, in rice exposed to Mn-toxicity, Mn was predominantly accumulated in leaves compared with roots [39]. The tolerance of plants to Mn is associated not only with low Mn uptake, but also with relatively little Mn translocation from roots to shoots [40,41]. Our results showed that under Mn-toxicity, *C. sinensis* plants accumulated more Mn in roots and less Mn in shoots than *C. grandis* ones, and that the concentration of Mn was lower in Mn-toxicity *C. sinensis* leaves than in *C. grandis* ones (Figure 2). This might contribute to the Mn-tolerance of *C. sinensis*. We isolated 122 differentially expressed TDFs from Mn-toxicity *C. grandis* leaves, which belong to different functional categories (Additional file 2 and Figure 7B), meaning that Mn-toxicity affected different physiological and biochemical pathways. In contrast, we only identified 15 differentially expressed TDFs from Mn-toxicity *C. sinensis* leaves (Additional file 3 and Figure 7A). Obviously, the transcript profile was less affected by Mn-toxicity in *C. sinensis* leaves compared to *C. grandis* ones, which may be associated with the less Mn accumulation in Mn-toxicity *C. sinensis* leaves (Figure 2C and G). These data also support above inference that *C. sinensis* is more tolerant to Mn-toxicity than *C. grandis*.

### Genes involved in biological regulation and signal transduction

Protein phosphorylation, a versatile post-translational modification (PTM), is involved in almost all plant signal pathways and plays important roles in regulation of abiotic stress responses [42]. Phosphorylation and dephosphorylation of a protein often serve as an “on-and-off” switch in the regulation of cellular activities. For optimal regulation, kinases and phosphatases must strike a balance in any given cell [43]. As shown in Additional file 2, Mn-toxicity decreased the expression levels of genes involved in phosphorylation [leucine-rich receptor-like protein kinase (TDF #066-4), probable receptor-like protein kinase (TDF #104-5), Ser/Thr protein kinase isolog (TDF #170-1), VH1-interacting kinase (TDF #199-1), calcium-dependent protein kinase 1-like (TDF #165-2) and OBP3-responsive gene 1 (TDF #238-1) genes] and dephosphorylation [protein phosphatase 2a, regulatory subunit, putative (TDF #044–1)] except for increased expression of a mitogen-activated protein kinase 1 (MAPK 1, TDF #089–1) gene in *C. grandis* leaves. This means that the balance between phosphorylation and dephosphorylation was upset and phosphorylation of some proteins might be impaired in Mn-toxicity *C. grandis* ones. MAPK cascades are important signal modules that convert signals generated at the receptors/sensors to appropriate cellular responses [44]. Increasing evidence demonstrates the role of MAPK signal in different heavy metal stresses for different plant species [45]. The observed higher expression level of *MAPK 1* (TDF #089–1) in Mn-toxicity leaves agrees with the previous reports that excess copper (Cu), cadmium (Cd) and mercury (Hg) led to the activation of a novel MAPK gene *OsMSRMK2* from Japonica-type rice [46], and that MAPK pathways were activated by excess Cu and Cd in *M. sativa* seedlings [47]. Thus, MAPK cascade might play a role in the responses of plants to Mn-toxicity. In contrast to *C. grandis*, VH1-interacting kinase (TDF #199a) gene in *C. sinensis* leaves was induced by Mn-toxicity (Additional file 3).

MAPKs are able to phosphorylate different substrates in different cellular compartments, including transcription factors (TFs) in the nucleus [29]. Thus, genes related to TFs might be affected in Mn-toxicity *C. grandis* leaves due to altered expression level of *MAPK 1* (TDF #089-1) gene (Additional file 2). As expected, Mn-toxicity increased the expression levels of genes encoding TF jumonji domain-containing protein (TDF #09-2) and Myb family transcription factor (TDF #232-1), and decreased the expression levels of genes encoding TF ILR3 (TDF #105-2), C3H4 type zinc finger protein (TDF #156-2), putative TF (TDF #045-2) and DNA-binding storekeeper protein-related transcriptional regulator (TDF #131-1) in *C. grandis* leaves (Additional file 2). Jumonji C (jmjC) domain-containing proteins have been shown to function as demethylases and to involve in chromatin structure and gene expression [48]. Recently, Govind et al. [49] showed that two *Jumonji TFs* (*Jumonji like TF* and *TF jumonji domain-containing protein*) and other genes (*Lea5, HSP20* and *HSP70*) were induced in drought-stressed peanut (*Arachis hypogaea*) plants, which agrees with our results that Mn-toxicity *C. grandis* leaves had higher mRNA levels of gene encoding TF jumonji domain-containing protein (TDF #09-2, Additional file 2). They also found that silencing of *Jumonji (JMJC)* made the transgenic tobacco (*Nicotiana benthamiana*) plants more tolerant to drought, while down-regulation of *HSP70* resulted in susceptibility [49]. Rampey et al. [50] reported that *ilr3-1 Arabidopsis* seedlings were less sensitive than wild type to Mn-toxicity. ILR3 is a basic helix–loop–helix type TF, which seems to regulate metal homeostasis in part through the action of putative Fe/Mn CCC1-like (VIT1-like) transporters. Therefore, the down-regulation of *ILR3* in Mn-toxicity *C. grandis* leaves might be an adaptive response. Like *C. grandis*, the expression of DNA-binding storekeeper protein-related transcriptional regulator (TDF #131a) was inhibited in *C. sinensis* leaves. *ILR3* (TDF #105b), however, was up-regulated in Mn-toxicity *C. sinensis* leaves (Additional file 3).

Calcium (Ca) is a secondary messenger and has been suggested to participate in heavy metal signal [29]. Indeed, Ca concentration in runner bean (*Phaseolus coccineus*) cells greatly increased in response to Cd stress [51]. Ca may interact with calmodulin to propagate the signal and ultimately to regulate downstream genes involved in heavy metal transport, metabolism, and tolerance [52]. In this study, Mn-toxicity decreased the expression levels of Ca-dependent protein kinase 1-like (TDF #165-2), calmodulin-binding transcription activator 5 (TDF# 102) and Ca^2+^-binding protein (TDF #186-2) genes in *C. grandis* leaves, and increased the expression levels of Ca-binding EF-hand domain-containing protein (TDF #153-3) gene in *C. grandis* leaves and calmodulin-binding transcription activator 5 (TDF #100b) in *C. sinensis* ones (Additional file 2 and Additional file 3). Thus, genes related to Ca^2+^ signal might be involved in response to Mn-toxicity. Busov et al. [53] characterized a 5NG4 gene from juvenile loblolly pine shoots, which was highly and specifically induced by auxin prior to adventitious root formation. Toxicity of Mn, on the other hand, led to auxin deficiency caused by activation of indole-acetic acid oxidase under excess Mn [20]. This supports our data that auxin-induced protein 5NG4 gene (TDF #233a) was down-regulated in *C. sinensis* leaves (Additional file 3). Similarly, the transcript abundance of auxin-response factor (TDF #111-2) in *C. grandis* leaves also decreased in response to Mn toxicity (Additional file 2). However, Mn-toxicity increased *5NG4* mRNA level in *C. grandis* ones (TDF #233-1, Additional file 2).

Transducin/WD-40 repeat-containing protein is a G protein involved in a wide range of functions that include signal transduction, transcription and stress-tolerant function. It was up-regulated by excess Cu in germinating rice seeds [54,55], but was down-regulated by Mn-toxicity in *C. grandis* leaves (TDF #085-3, Additional file 2).

VQ motif-containing proteins play important roles in plant growth, development and defense responses. Loss-of function mutants and/or overexpression lines for the VQ genes are altered in seed size, tolerance to abiotic stress, or resistance to pathogen infection [56,57]. Liu et al. [56] reported that transgenic *Arabidopsis* plants overexpressing the *AtARVQ1*, a gene encoding a plant-specific VQ motif-containing protein, were more resistant to arsenate stress. Recently, Cheng et al. [58] showed that plant VQ motif-containing proteins played critical roles in the network of WRKY-mediated gene expression. We found that under Mn-toxicity, the expression level of VQ genes (TDF #061-2) was decreased in *C. grandis*, which is in agreement with the previous report that *AtARVQ1* expression was strongly down-regulated by arsenate stress [56].

Zhang et al. [59] observed that *A. thaliana Fes1A* could prevent cytosolic HSP70 degradation. Thus, the observed lower mRNA level of HSP70 nucleotide exchange factor *fes1* (TDF #235-2, Additional file 2) in Mn-toxicity *C. grandis* leaves agrees with the results that the expression levels of HSP70 (TDF #237-6) and stromal 70 kDa heat shock-related protein, chloroplastic-like gene (TDF #156-1) in *C. grandis* ones decreased in response to Mn-toxicity (Additional file 2). Similar result has been obtained on B-deficient *C. sinensis* roots [60].

Phosphatidylinositol (PtdIns) 4-kinase catalyzes the phosphorylation of PtdIns in the D-4 position of the inositol ring. This is the committed step in the synthetic pathway leading to PtdIns 4,5-bisphosphate (PtdIns 4,5-P_2_). Apart from this function in signal transduction, both PtdIns 4-P and PtdIns 4,5-P_2_ appear to have other cellular functions (such as regulating cytoskeletal architecture, acting as enzyme effectors, or acting as components of vesicular fusion [61]. We found that the expression level of PtdIns 4-kinase type 2-beta (TDF #099-2) in *C. grandis* leaves decreased in response to Mn-toxicity (Additional file 2), which disagrees with Mn-stimulated PtdIns 4-kinase from *Dunaliella parva*[62].

Based on these results above mentioned, we conclude that the responses of citrus plants to Mn-toxicity might be regulated in multiple signal pathways.

### Genes involved in carbohydrate and energy metabolism

Thirteen genes in *C. grandis* leaves and two genes in *C. sinensis* ones related to carbohydrate and energy metabolism were altered under Mn-toxicity (Additional file 2 and Additional file 3, Figure 7). ATP synthase is an important enzyme that provides energy for the cell to use through the synthesis of ATP. Hamilton et al. [63] showed that exposure of wheat to Al increased mitochondrial F_1_F_0_-ATPase activity only in the Al-tolerant wheat variety, and concluded that the Al-induced increase in ATP synthase activity was an adaptive response involved in Al tolerance. Similar to Al-toxicity, Mn-toxicity increased the expression levels of synthase subunit alpha (Atp 1) in *C. grandis* (TDF #065-1) and *C. sinensis* (TDF #65a) leaves and ATP synthase subunit alpha (TDF #01-1) in *C. grandis* leaves (Additional file 2 and Additional file 3). Aconitate hydrolyase (AH) involved in glycolysis catalyses the interconversion of citrate and isocitrate, *via* a *cis*-aconitate. Our finding that the expression of AH 2 (TDF #116-1, Additional file 2) was induced in Mn-toxicity *C. grandis* leaves means that its activity was enhanced by Mn-toxicity, which is in agreement with the previous report that AH was up-regulated by Cd stress [64]. In addition, Mn-toxicity also increased the expression levels of NADPH-ferrihemoprotein reductase (TDF #178-1) and cytochrome (Cyt) c biogenesis orf256 (TDF #236-1) genes related to respiration in *C. grandis* leaves (Additional file 2). Overall, the metabolic pathways related to ATP synthesis and reducing power production might be activated in Mn-stressed leaves to yield more energy needed to meet the high-energy demand of stressed cell. By contrast, the mRNA level of gene encoding 2-phospho-D-glycerate hydrolase (also known as enolase, TDF #244-1), an essential and ubiquitous glycolytic enzyme responsible for the catalysis of the conversion of 2-phosphoglycerate (2-PG) to phospho*enol*pyruvate (PEP), was down-regulated in Mn-toxicity *C. grandis* leaves (Additional file 2). This agrees with the previous report that the abundance of enolase in *Agrostis stolonifera* leaves was decreased after 10 d of heat stress [65]. Ferreira et al. in *Populus euphratica* leaves observed that the level of enolase was transiently increased after 30 h of heat stress, followed by a decrease after 54 h of heat stress [66]. However, other studies showed that leaf enolase was induced in various plants by different abiotic stresses, such as water stress in maize [67], cold stress in tobacco [68], salt stress in ice plant (*Mesembryanfhemum crysfallinum*) [69] and Cd-stress in *Phytolacca americana*[70]. Like to enolase, the expression level of gene encoding Cyt P450 (TDF #197-1), an enzyme typically catalyzing the reaction: RH + O_2_ + NADPH + H^+^ → ROH + H_2_O + NADP^+^, was reduced in Mn-toxicity *C. grandis* leaves (Additional file 2), which might contribute to maintaining NADPH homeostasis by decreasing its utilization. However, the expression of Cyt P450, family 96, subfamily A, polypeptide 9 (TDF #029a) gene in *C. sinsnsis* leaves was induced by Mn-toxicity (Additional file 3), which is in agreement with the report that at least 5 of the 29 *Cyt P450s* were induced in *Arabidopsis* by abiotic and biotic stress including *Alternaria brassicicola* or *Alternaria alternata*, paraquat, rose bengal, UV-C stress, heavy metal stress (CuSO_4_), mechanical wounding, drought, high salinity, low temperature or hormones [71]. Transgenic tobacco and potato plants expressing *Cyt P450* tolerated better oxidative stress after herbicide treatment [72]. Therefore, the observed higher mRNA level of *Cyt P450, family 96, subfamily A, polypeptide 9* might be advantegous to Mn-toxicity tolerance of *C. sinensis* plants.

Thioredoxins (Trxs) are small proteins, which are involved in the cell redox regulation. Trx *m*, a chloroplastic protein, preferentially activates NADP-malate dehydrogenase (NADP-MDH), a key enzyme involved in carbon fixation and sugar biosynthesis during photosynthesis. Historically the m-type Trxs were so named because they were able to activate NADP-MDH [73]. The activity of NADP-MDH might be down-regulated in Mn-toxicity *C. grandis* leaves due to lower mRNA levels of Trx *m* (TDF #058-2) and Trx *m*4 (TDF #085-2) (Additional file 2). Evidence shows that Trx *m*1, *m*2, and *m*4 could act as antioxidants, i.e. possibly by serving as hydrogen donors for a Trx-dependent peroxidase [74].

Trehalose-6-phosphate synthase (TPS) is a key enzyme for synthesizing trehalose, which plays an important role in abiotic stress tolerance of plants [75]. The observed lower level of *TPS* (TDF #013-3) in Mn-xicity *C. grandis* leaves (Additional file 2) indicates that Mn-toxicity might decrease leaf concentration of trehalose, thus lowering the stress tolerance.

ATP sulfurylase (ATPS) activity were enhanced by sulphur (S) deprivation and reduced after resupply of SO_4_^2–^[76]. Heiss et al. [77] reported that Cd stress strongly increased the expression levels of ATPS and 5’-adenylysulfate reductase in roots and leaves of 6-week-old *Brassica juncea*, accompanied by enhanced cysteine concentration in roots and leaves, and concluded that roots and leaves responded to Cd stress with a coordinative up-regulation of several S assimilation enzymes to meet an increased demand for cysteine during phytochelatin synthesis. However, the expression of *ATPS* (TDF #168-1) was down-regulated in Mn-toxicity *C. grandis* leaves (Additional file 2).

UDP-glycosyltransferases (UGTs), which glycosylate a broad array of aglycones, including plant hormones, all major classes of plant secondary metabolites, and xenobiotics such as herbicides, play an important role for the stabilization, enhancement of water solubility and detoxification of natural products [78]. Cytokinins can be glucosylated to form O-glucosides and N-glucosides. The glycoconjugates are inactive and are considered to play a role in hormonal homeostasis. Hou et al. [79] showed that UGT76C1 and UGT76C2 (two members of group H) recognized all cytokinins and glucosylated them at the *N*^7^ and *N*^9^ positions. Veselov et al. [21] reported that Cd stress sharply lowered the concentration of cytokinin in wheat roots and shoots caused by elevated activity of cytokinin oxidase. Obviously, the down-regulation of UDP-glycosyltransferase 76F1-like (one member of group H, TDF #185-4) gene in Mn-toxicity *C. grandis* leaves (Additional file 2) is advantageous to cytokinin homeostasis under Mn-toxicity if leaf concentration of cytokinin decreased in response to Mn-toxicity.

### Genes involved in nucleic acid metabolism

Evidence shows that heavy metals inhibit plant nucleic acid metabolism [80,81]. As expected, the expression levels of genes encoding THO complex, subunit 5 (TDF #134-2), DNA polymerase phi subunit (TDF #139-1), histone H4 (TDF #165-1), DNA (cytosine-5)- methyltransferase DRM2-like (TDF #200-1) and luc7-like protein 3-like (TDF #200-2) in *C. grandis* leaves (Additional file 2) and genes encoding THO complex, subunit 5 (TDF #134b) and histone H4 (TDF #165a) in *C. sinensis* ones (Additional file 3) decreased in response to Mn-toxicity, indicating that leaf nucleic acid metabolism might be impaired by Mn-toxicity.

### Genes involved in protein metabolism

Mn-toxicity has been demonstrated to affect protein metabolism in plants [12]. We found that the expression levels of three ribosomal genes encoding ribosomal protein S3 (TDF #06-1), ribosomal protein S8 (TDF #097-1) and 60S ribosomal protein L2, mitochondrial-like (TDF #134-1), which are involved in mature ribosome assembly and translation processes, were down-regulated in Mn-toxicity *C. grandis* leaves (Additional file 2), while only one ribosomal gene encoding 60S ribosomal protein L2, mitochondrial-like (TDF #134a) in *C. sinensis* leaves was down-regulated by Mn-toxicity (Additional file 3). This indicates that the biosynthesis of protein in Mn-toxicity *C. grandis* leaves might be impaired more severe than in Mn-toxicity *C. sinensis* ones, which is in agreement with our data that Mn-toxicity decreased the concentration of total soluble protein in *C. grandis* leaves, but did not affect its concentration in *C. sinensis* ones (Figure 4A). However, the expression of 30S ribosomal protein S13 gene (TDF #140-1) was induced in Mn-toxicity *C. grandis* leaves (Additional file 2).

Translational control of protein synthesis depends on numerous eukaryotic initiation factors (eIFs). Our finding that the expression of gene encoding similar to translation initiation factor IF2 (TDF #011-2) was down-regulated in Mn-toxicity *C. grandis* leaves (Additional file 2) agrees with our previous report that the abundances of eIF4B1, eIF3 subunit and eIF3G1 in *C. sinensis* roots decreased in response to B-deficiency [60]. However, salt and heavy metal induced the accumulation of rice eIF5A-1 and eIF5A-2 mRNAs in rice cells [82]. Elongation factors whose expressions have been demonstrated to correlate to the high rate of protein synthesis in developing plant tissues [83] facilitate translational elongation, from the formation of the first peptide bond to the formation of the last one in the ribosome. Guo et al. [84] isolated a translation elongation factor 2-like protein gene from a cold defective *Arabidopsis* mutant, *los 1–1*. The *los1-1* mutant plants were impaired in protein synthesis under cold stress. We found that the expression level of one gene encoding translation elongation factor-1 alpha, partial (TDF #181-2) in *C. grandis* leaves decreased in response to Mn-toxicity (Additional file 2), which agrees with the previous results obtained on drought-stressed soybean nodules [85], anoxia maize roots [86], and B-deficient roots of *C. sinensis*[60], *Brassica napus*[87] and *Lupinus albus*[88]. However, the abundances of chloroplast translational elongation factor Tu and elongation factor P in rice leaves increased in response to Cd stress [89]. Thus, it appears that the influence of abiotic stress on translation elongation factors depends on plant species and kinds of stresses. In addition, the expression level of gene encoding tetratricopeptide repeat-containing protein (TDF #090-1) involved in protein synthesis was down-regulated in Mn-toxicity *C. grandis* leaves (Additional file 2). These results further demonstrate that protein biosynthesis is impaired in Mn-toxicity *C. grandis* leaves.

Mn-toxicity increased the expression levels of genes encoding ATP-dependent Clp protease (TDF #245-1), cysteine proteinase (TDF #044-2) and xylem cysteine proteinase 2 (TDF #098-2) in *C. grandis* leaves (Additional file 2). This indicates that the hydrolysis of some proteins might be up-regulated in Mn-toxicity leaves, thus decreasing leaf concentration of total soluble protein. This is also supported by our data that Mn-toxicity leaves had lower concentration of total soluble protein (Figure 4A). However, the expression levels of genes encoding carboxyl-terminal peptidase (TDF #104-6), papain family cysteine protease (TDF #107-1), cathepsin B-like cysteine proteinase like protein (TDF #233-3) and α/β-hydrolase-like protein (TDF #221-1) decreased in response to Mn-toxicity (Additional file 2).

Like other PTMS (such as phosphorylation), ubiquitination, which serves as a versatile PTM, plays a key role in regulating plant response to abiotic stresses [90]. The up-regulation of genes encoding MND1-interacting protein 1 (TDF #148-1) and ubiquitin-conjugating enzyme E2 10 (TDF #240-1) in Mn-toxicity *C. grandis* leaves (Additional file 2) might be an adaptive response to Mn-toxicity. However, the expression levels of ubiquitin-correlative genes such as ubiquitin-protein ligase (TDF #03-2) and BTB and MATH domain-containing protein (TDF #138-2) in *C. grandis* leaves decreased in response to Mn-toxicity (Additional file 2), meaning that the ubiquitination of some proteins might be impaired in Mn-toxicity *C. grandis* leaves.

Three genes [chorismate synthase (TDF #247-1), cystathionine β-synthase (CBS) domain-containing protein (TDF #216-3) and 2-oxoglutarate (2OG) and Fe(II)-dependent oxygenase-like protein (TDF #061-3) ] involved in amino acid metabolism was down-regulated in Mn-toxicity *C. grandis* leaves (Additional file 2). This means that the biosynthesis of amino acids might be impaired in Mn-toxicity *C. grandis* leaves. Chorismate synthase catalyzes the last of the seven steps in the shikimate pathway which is used in prokaryotes, fungi and plants for the biosynthesis of aromatic amino acids [91]. CBS catalyzes the first step of the transsulfuration pathway from homocysteine to cystathionine. Jung et al. [92] observed that CBSX2 directly modulated Trx in chloroplasts, which affected the level of H_2_O_2_ and, consequently, the expression of the genes involved in secondary cell-wall thickening, concluding that CBSX2 protein played a critical role in thickening of the secondary cell walls of the endothecium during anther dehiscence in *Arabidopsis*. Transgenic tobacco plants overexpressing *OsCBSX4* isolated from rice displayed enhanced tolerance to salinity, heavy metal, and oxidative stress [93]. This observed lower expression level of gene encoding CBS domain-containing protein (TDF #216-3) in *C. grandis* leaves (Additional file 2) disagrees with our previous data that the abundance of CBS family protein in *C. sinensis* roots increased in response to B-deficiency [60].

### Genes involved in lipid metabolism

Lecithin-cholesterol acyltransferase (LCAT) catalyzes the transacylation of acyl groups from phospholipids to sterols in mammals and yeast. Sterol acylation is an essential process of sterol homeostasis in eukaryotic cells. In *Arabidopsis*, phospholipid sterol acyltransferase 1 (PSAT1), which displays homology with the mammalian LCAT, catalyzes a phospholipid-dependent (acyl-CoA-independent) formation of sterol esters [94]. Recent work with *Arabidopsis* showed that sterol ester concentration decreased in leaves of *psat1-1* or *psat1-2* mutants accompanied by an early leaf senescence phenotype, suggesting a major contribution of the *PSAT1* in maintaining both free sterol homeostasis in plant cell membranes and leaf viability during developmental aging [95]. The up-regulation of lecithin-cholesterol acyltransferase-like 1 (TDF #153-2, Additional file 2) in Mn-toxicity *C. grandis* leaves might enhance the leaf concentration of sterol ester, thus preventing leaf senescence.

### Genes involved in cell wall metabolism

Our results showed that the expression levels of genes [α-1, 2-fucosyltransferase (TDF #037-3), caffeic acid O-methyltransferase (TDF #080-1), O-fucosyltransferase family protein (TDF #069-8), cellulose synthase-like protein (TDF #044-4), protein SAH7 (TDF #05-1), 4-coumarate-CoA ligase 3 (TDF #151-1) and CBS domain-containing protein (TDF #216-3)] involved in cell wall biosynthesis were down-regulated in Mn-toxicity *C. grandis* leaves (Additional file 2). In addition, the mRNA levels of gene encoding glycoside hydrolase family 28 protein (TDF #043-1), which catalyze the hydrolytic cleavage of the pectin and gene encoding cell wall-associated hydrolase (TDF #242-1) were up-regulated in Mn-toxicity *C. grandis* leaves (Additional file 2). Therefore, the formation of cell wall might be impaired in Mn-toxicity leaves. However, the expression of gene encoding protein trichome birefringence-like 39 (TDF #158-3) was induced in Mn-toxicity *C. grandis* leaves (Additional file 2).

### Genes involved in stress responses

Mn-toxicity has been demonstrated to stimulate ROS production in plants [12,14,16]. Since Mn-toxicity did not affect the concentration of MDA in citrus leaves (Figure 4B), some protective antioxidant enzymes should be up-regulated to meet the increased requirement for scavenging ROS. Our results showed that the expression of gene encoding catalase (CAT, TDF #103-2), an enzyme involved in scavenging H_2_O_2_, was induced in Mn-toxicity *C. grandis* leaves (Additional file 2), which agrees with the previous report that Mn-toxicity *C. grandis* leaves had increased specific activity of CAT and similar leaf area-based activity of CAT [8]. Besides ROS scavenger enzymes, this detoxification mechanism also involves “house-keeping” enzymes. The family of Nudix hydrolases (NUDXs), which catalyze the hydrolytic breakdown of nucleoside diphosphates linked to some other moieties such as a phosphate, sugar or nucleoside, are one of these “house-keeping” enzyme families [96]. Ogawa et al. [97] showed that AtNUDX19, a chloroplastic AtNUDX, played an important role in modulation of the NADH and/or NADPH pools through the hydrolysis of NAD(P)H to reduced nicotinamide mononucleotide (NMNH) in *Arabidopsis* chloroplasts. Transgenic *Arabidopsis* plants overexpressing *AtNUDX2* and *AtNUDX7* displayed enhanced tolerance to oxidative stress, resulting from the maintenance of NAD^+^ and ATP levels by nucleotide recycling of free ADP-ribose molecules [98]. The higher expression level of *NUDX19* (TDF #160-1) in *C. grandis* leaves (Additional file 2) agrees with the previous reports that the abundance of NUDX homolog 3 in *C. sinensis* roots increased in response to B-deficiency [60] and *Chrysanthemum lavandulifolium ClNUDX1*, *ClNUDX3*, *ClNUDX7* and *ClNUDX8* were induced by salt, drought, heat, and cold stresses [99]. However, the expression levels of genes encoding monodehydroascorbate reductase (MDAR, TDF #098-1), peroxidase 42 (TDF #104-1), glutathione S-transferase (GST) Tau2 (TDF #227-1), NADP-dependent alkenal double bond reductase P2 (TDF #130-2), Trx *m* (TDF #058-2), TRx *m*4, chloroplastic-like (TDF #085-2), and CBS domain-containing protein (TDF #216-3) related to oxidative defense in *C. grandis* leaves decreased in response to Mn-toxicity (Additional file 2). It is noteworthy that Mn-toxicity *C. grandis* leaves had higher specific activity of MDAR and leaf area-based activity of MDAR compared with controls [8]. The discrepancy between the expression level of *MDAR* and the activity of the corresponding enzyme indicates that PTMs might affect MDAR activity.

**Figure 9:**
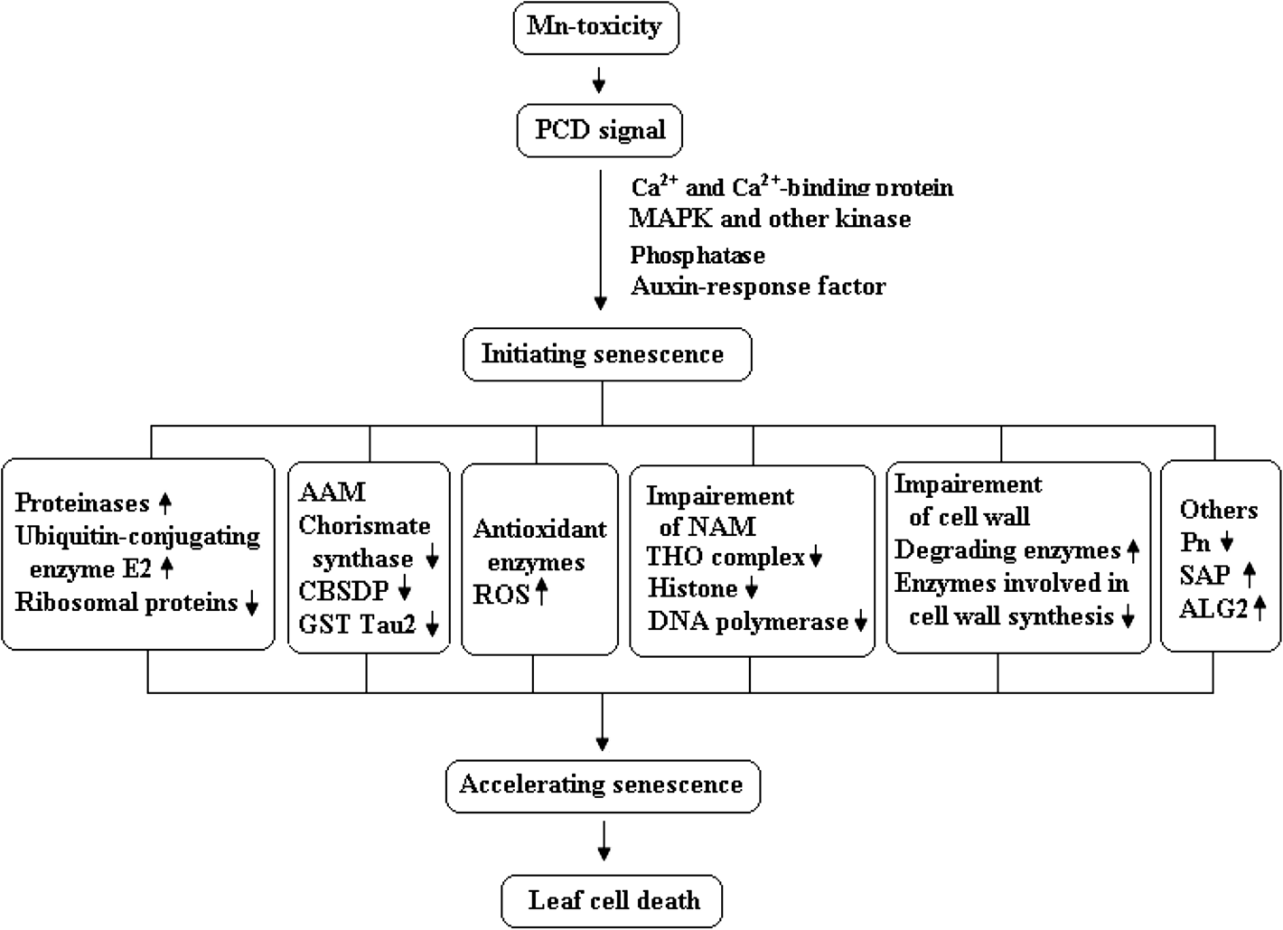
**The potential regulatory network of Mn**-**toxicity**-**induced senescene in *Citrus grandis *leaves.** AAM: Amino acid metabolism; ALG2: Apoptosis linked gene 2 (ALG2)-interacting protein X; CBSDP: Cystathionine β-synthase (CBS) domain-containing protein; GST: Glutathione S-transferase; MAPK: mitogen-activated protein kinase; NAM: Nucleic acid metabolism; PCD: Programmed cell death; Pn: Photosynthesis; ROS: Reactive oxygen; SAP: Senescence-associated protein; ↑: Up-regulation; **↓**: Down-regulation.

Senescence is a genetically programmed decline in various cellular processes and involves in the hydrolysis of macromolecules such as proteins and lipids. It is governed by the developmental age and is induced or enhanced by biotic and abiotic stresses. Generally in plants the term senescence and programmed cell death (PCD) denote the processes that initiate the programmed death of individual cell. The senescence can be considered as one of the examples for PCD. As expected, the expression level of one gene encoding for putative senescence-associated protein (TDF #164-4) was up-regulated by Mn-toxicity in the less Mn-tolerant *C. grandis* leaves (Additional file 2). This agrees with the previous report that the gene was highly differentially expressed only in the heat-sensitive fescue (*Festuca* sp.) genotype at 44°C [100]. In addition, the expression levels of apoptosis linked genes [xylem cysteine proteinase 2 (TDF #098-2), cysteine proteinase (TDF #044-2) and ALG2-interacting protein X (TDF #054-1) genes] were enhanced in Mn-toxicity *C. grandis* leaves (Additional file 2). Thus, it is reasonable to assume that the Mn-toxicity sensitive *C. grandis* plants were under great stress and leaf senescence was accelerated, which might contribute to plant survival by using more metabolites through glycolysis, and protein and lipid degradation. As shown in Figure 9, the Mn-toxicity-induced senescence in *C. grandis* leaves was a very complicated process.

HSPs/chaperones have been demonstrated to play a key role in protecting plants against heavy metal stress [64]. The observed lower expression levels of genes encoding heat shock protein-related, partial (TDF #158-2), heat shock protein 60-3A (TDF #160-5), HSP70 (TDF #237-6) and stromal 70 kDa heat shock-related protein, chloroplastic-likes (TDF #156-1) in Mn-toxicity *C. grandis* leaves (Additional file 2) indicate decreased protein synthesis in these leaves, as indicated by decreased leaf concentration of total soluble proteins (Figure 4A). This is similar to the previous report that the abundance of HSP70 was enhanced in leaves of high Cd-accumulating soybean cultivar, but was lowered in low Cd-accumulating one [101].

One crucial adaptive mechanism of plants to P-deficiency is the immediate cleavage of phosphate (Pi) from phosphorylated substrates. Phosphoethanolamine/phosphocholine phosphatase (PPsPase) may release Pi from organic to maintain Pi homeostasis of plant cells [102]. The down-regulation of PPsPase, putative gene (TDF #198-1) in *C. grandis* leaves (Additional file 2) might decrease the release of Pi from phosphorylated substrates, thus lowering leaf P concentration (Figure 3A). This agrees with the previous report that Mn-toxicity decreased P concentration in tea leaves [17].

### Genes involved in cell transport

Eight genes in *C. grandis* leaves and one gene in *C. sinensis* ones were regulated by Mn-toxicity (Additional file 2 and Additional file 3, Figure 7). We found that the expression of *citrus sucrose transporter 1* (TDF #073-1, Additional file 2) was induced in Mn-toxicity *C. grandis* leaves, which is in agreement with the previous report that the gene strongly expressed in source, sugar exporting organs [103], because Mn-toxicity increased or did not affect the concentrations of non-structural carbohydrates in *C. grandis* leaves [8]. The up-regulation of *citrus sucrose transporter1* might be helpful to decrease sugar accumulation in Mn-toxicity leaves, which might in turn lead to feedback suppression of CO_2_ assimilation [104].

One of strategies of plants to deal with heavy metals is to transport them out of the cells, thereby removing them from the cytosol. Kim et al. [105] observed that transgenic *A. thaliana* plants overexpressing the ABC transporter *AtPDR8* were more resistant to Cd or lead (Pb) and displayed lower Cd concentration in roots and shoots than wild-type plants, while *AtPDR8* RNAi transgenic plants and T-DNA insertion lines were more sensitive to Cd or Pb and had higher Cd concentration, concluding that the ABC transporter AtPDR8 is a Cd extrusion pump conferring heavy metal resistance. The down-regulation of genes encoding ABC-transporter-like protein (TDF #208-1) and ABC transporter family protein (TDF #248-1) in *C. grandis* leaves (Additional file 2) indicates that the extrusion of Mn from leaves might be lessened in response to Mn-toxicity, which might be one of the causes that Mn-toxicity *C. grandis* leaves accumulated more Mn than *C. sinensis* ones (Figure 2G).

Evidence has shown that CorA-like proteins may represent the major transport systems in eukaryotes such as yeast, animals, and plants [106,107]. The down-regulation of Mg transporter CorA-like protein gene in Mn-toxicity *C. grandis* (TDF #234-2, Additional file 2) and *C. sinensis* (TDF #234b, Additional file 3) leaves might reduce Mg transport, hence decreasing leaf Mg concentration. This agrees with the previous report that Mn-toxicity decreased leaf concentration of Mg [17]. Interestingly, Mg concentration was decreased by Mn-toxicity only in *C. grandis* leaves, but was not significantly affected in *C. sinensis* leaves (Figure 3B). This means that leaf Mg concentration is also regulated by other factors.

Plant cyclic nucleotide gated channels (CNGCs), which comprise a large gene family in *Arabidopsis*, have been proposed to be involved in multiple plant physiological processes including plant growth and heavy metal toxicity tolerance [108]. Evidence suggests that CNGCs paly a role in heavy metal homeostasis, for example, in tobacco, overexpression of tobacco *CNGC* (*NtCBP4*) led to hypersensitivity to Pb [109]. However, Sunkar et al. [110] showed that transgenic tobacco plants overexpressing a truncated *NtCBP4* exhibited improved tolerance to Pb^2+^ and decreased uptake of this metal ion. We found that the expression of cyclic nucleotide gated channel 9 gene (TDF #216-2) was down-regulated in Mn-toxicity *C. grandis* leaves (Additional file 2), suggesting that the gene might be involved in the response to Mn-toxicity.

Our results showed that the expression of gene encoding protease inhibitor/seed storage/lipid transfer protein (LTP) family protein (TDF #242-2) was down-regulated in Mn-toxicity *C. grandis* leaves (Additional file 2), indicating that the exchange of lipids between membranes might be decreased.

## Conclusions

Our results clearly demonstrated that *C. sinensis* was more tolerant to Mn-toxicity than *C. grandis*. Under Mn-toxicity, *C. sinensis* plants accumulated more Mn in roots and less Mn in shoots (leaves) than *C. grandis* ones, and that the leaf concentration of Mn was lower in the former. This might contribute to the Mn-tolerance of *C. sinensis*. In this study, we first used the cDNA-AFLP technique to compare the mRNA levels of genes from control and Mn-toxicity leaves of two citrus species differing in Mn-tolerance. In Mn-toxicity *C. grandis* leaves, 42 up-regulated and 80 down-regulated genes were isolated, while only seven up-regulated and eight down-regulated genes were identified in Mn-toxicity *C. sinensis* ones. Obviously, Mn-toxicity affected gene expression far less in *C. sinensis* leaves than in *C. grandis* ones, which might be associated with less leaf Mn concentration in Mn-toxicity *C. grandis* leaves. cDNA-AFLP analysis suggests that the responses of *C. grandis* leaves to Mn-toxicity might include following several aspects: (1) accelerating leaf senescence; (2) activating the metabolic pathway related to ATPase synthesis and reducing power production; (3) decreasing cell transport; (4) inhibiting protein and nucleic acid metabolisms; (5) impairing the formation of cell wall; and (6) triggering multiple signal transduction pathways. We also identified many new Mn-toxicity-responsive genes involved in biological and signal transduction (i.e. VH1-interacting kinase, OBP3-responsive gene 1, transcription factor IL), carbohydrate metabolism (i.e. NADPH-ferrihemoprotein reductase, trehalose-6-phosphate synthase), protein metabolism (i.e. MND1-interacting protein 1, chorismate synthase), stress responses (i. e. Nudix hydrolase 19, ALG2-interacting protein X) and cell transport (i.e. cyclic nucleotide gated channel 9). Further studies will elucidate the roles of these genes in response to Mn-toxicity, which will help me to design Mn-tolerant transgenic crops.

## Methods

### Plant culture, Mn treatments and sampling

The study was conducted from April to December, 2012 at Fujian Agriculture and Forestry University (FAFU). Plant culture, Mn treatments, and sampling were performed according to Li et al. [8]. Briefly, 6-week-old seedlings of ‘Xuegan’ (*Citrus sinensis*) and ‘Sour pummelo’ (*Citrus grandis*) were transplanted to 6 L pots containing sand. Seedlings, two per pot, were grown in a greenhouse under natural photoperiod at FAFU. Each pot was supplied with 500 mL of nutrient solution every two day. The nutrient solution contained the following macronutrients (in mM): KNO_3_, 1.25; Ca(NO_3_)_2_, 1; (NH_4_)H_2_PO_4_, 0.25; MgSO_4_, 0.5; and micronutrients (in μM): H_3_BO_3_, 10; ZnCl_2_, 2; CuSO_4_, 0.5; (NH_4_)_6_Mo_7_O_24_, 0.065; MnSO_4_, 2; Fe-EDTA, 20. Eight weeks after transplanting, each pot was supplied every other day until dripping with nutrient solution (approximately 500 mL) containing 2 μM (control) or 600 μM (Mn-toxicity) MnSO_4_ for 17 weeks. At the end of the experiment, fully expanded leaves from different replicates and treatments were used for all the measurements. Leaves were collected at noon under full sun and immediately frozen in liquid nitrogen and were stored at −80°C until extraction.

### Measurements of root and shoot DW and determination of Mn, Mg and P

Ten plants per treatment from different pots were harvested and divided into their parts (roots, and shoots). The plant parts were then dried at 70°C for 48 h and the DW mesured.

Root, stem and leaf Mn concentration and leaf Mg concentration were assayed by inductively coupled plasma (ICP) emission spectrometry after microwave digestion with HNO_3_[111]. Leaf P concentration was measured according to Ames [112]. There were four replicates per treatment (one leaf per replicate, one leaf per plant).

### Determination of Chl, total soluble protein and MDA in leaves

Leaf Chl a+b, Chl a and Chl b were assayed according to Lichtenthaler [113]. Briefly, 2 frozen leaf discs (0.608 cm^2^ in size) were extracted with 8 mL of 80% (v/v) acetone for 24 h in the dark. The extracts were determined using Libra S22 ultraviolet–visible spectrophotometer (Biochrom Ltd., Cambridge, UK). Leaf total soluble protein was extracted with 50 mM Na_2_HPO_4_-KH_2_PO_4_ (pH 7.0) and 5% (w/v) insoluble polyvinylpolypyrrolidone (PVPP), and determined according to Bradford [114] using bovine serum albumin (BSA) as standard. Extraction and determination of leaf MDA was performed according to Hodges et al. [115]. There were four replicates per treatment (one leaf per replicate, one leaf per plant).

### Leaf gas exchange measurements

Measurements were made with a CIARS-2 portable photosynthesis system (PP systems, Herts, UK) at ambient CO_2_ concentration under a controlled light intensity of 1000 μmol m^-2^ s^-1^ between 9:30 and 10:30 on a clear day. During measurements, leaf temperature and vapor pressure deficit (VPD) were 26.9 ± 1.1°C and 2.0 ± 0.1 kPa, respectively. There were five replicates per treatment (one leaf per replicate, one leaf per plant).

### RNA preparation and cDNA synthesis

Total RNA was extracted from 200–300 mg of the frozen leaves using Recalcirtant Plant Total RNA Extraction Kit (Centrifugal column type, Bioteke Corporation, China) according to manufacturer’s instructions. The integrity and quantity of total RNA was detected by 1% (w/v) agarose gel electrophoresis and spectrophotometer at 260 nm. First-strand cDNA was synthesized from 2 μg of total RNA using RevertAid™ First Strand cDNA Synthesis Kit (Thermo Scientific, Massachusetts, USA). Second cDNA strand was performed with *Escherichia coli* RNase H, *E. coli* DNA Ploymerase I and T4 DNA Polymerase (TaKaRa, China), and stoped with 0.25 M EDTA (pH 8.0) and 10% sodium dodecyl sulfate (SDS). The resulting double-stranded cDNA was purified using equal volume of phenol : chloroform : isoamyl alcohol (25 : 24 : 1). Five μL was checked using agarose gel electrophoresis in order to observe an expected smear between 100 bp and 1000 bp.

### cDNA-AFLP analysis

The cDNA-AFLP-based transcript profiling procedure was performed as described by Cao et al. [116] with some modifications. Double-stranded cDNA (600 ng) was digested with restriction enzymes: 5 U each of *Eco*R I (Thermo Scientific, Massachusetts, USA; 3 h at 37°C) and *Mse* I (*Tru*1I, Thermo Scientific, Massachusetts, USA; 3 h at 65°C). The resulting restricted fragments were ligated to adaptors (*EcoR* I, 0.2 μM forward primer: 5’- CTCGTAGACTGCGTACC-3’ and reverse primer: 3’- CATCTGACGCATGGTTAAP −5’; *Mse* I, 2 μM forward primer: 5’-GACGATGAGTCCTGAG-3’ and reverse primer: 3’-TACTCAGGACTCATP-5’) with T4-DNA ligase (Thermo Scientific, Massachusetts, USA) for 10–16 h at 16°C. Prior to ligation, the two adaptors were heated at 94°C for 3 min, followed by 65°C for 10 min, 37°C for 10 min and 25°C for 10 min. The resulting ligated products were pre-amplified with the corresponding preamplification premiers: *Eco*R I, 5’-GACTGCGATCCAATTC-3’ and *Mse* I, 5’-GATGAGTCCTGAGTAA-3’. From a 100-fold dilution of the pre-amplified samples, a 5 μL diluted sample was used for the selective amplification using 256 combinations of the following primers: 16 derivatives of *Eco*R I primers 5’-GACTGCGATCCAATTCEE-3’ and 16 derivatives of *Mse* I primers 5’-GATGAGTCCTGAGTAAMM-3’; where EE and MM represent AA, AT, AC, AG, TA, TC, TT, TG, CA, CT, CG, CC, GA, GC, GT and GG. The selective amplification products were separated on a 6% (w/v) polyacrylamide gel run at 50 W for 2.5 h. The gels were silver stained as described by Bassam et al. [117] to visualize the cDNA bands. Samples for cDNA-AFLP analysis were run in two replicates at least.

The TDFs of interests were selected based on their presence, absence or differential intensity and cut out with a scalpel, and incubated in 50 μL of dd H_2_O for 30 min in a boiling water bath, then centrifuged at 10000 revolutions per min at room temperature (Eppendorf 5418R, Hamburg, Germany). The supernatant was stored at −20°C for re-amplification. The eluted DNA was re-amplified by PCR using the same primer combinations. The re-amplified products representing the Mn-toxicity-responsive TDFs were checked on 1% (w/v) agarose gels, each band is isolated and eluted using DNA Agarose Gel Recovery Kit (Solarbio, China). Before being sequenced by BGI Technology Corporation (Shenzhen, China), these TDFs fragments were ligated to pGEM-T EASY vector according to usage information of pGEM®-T Easy Vector System I (Promega, USA), then transduced into *E. coli* (DH5α) competent cells using Ampicillin as the selecting agent. All sequences were input into the VecScreen (http://www.ncbi.nlm.nih.gov/tools/vecscreen/) to identify and remove all of the vector sequence. Homology of TDFs’ sequences was analyzed using the BLASTX and BLASTN searching engines (http://blast.ncbi.nlm.nih.gov/Blast.cgi). Their functional categories were assigned based on the analysis of information reported for each sequence by The Gene Ontology (http://www.geneontology.org/) and Uniprot (http://www.uniprot.org/).

### Quantitative RT-PCR (qRT-PCR) analysis

Total RNA was isolated from the frozen leaves of control and Mn-excess plants by TRIzol reagent (Invitrogen, Carlsbad, CA, USA). About 2.0 μg total RNA was used for first-strand cDNA synthesis using the RevertAid™ First-Strand cDNA Synthesis Kit (Thermo Scientific, Massachusetts, USA) following the manufacturer’s instructions. The resulting cDNA was diluted to 100 μL using Tris-EDTA buffer (10 mM Tris, 50 mM NaCl, 1 mM EDTA, pH 7.8). Specific primers were designed from the sequences of 15 singleton TDFs using Primer Primier Version 5.0 (PREMIER Biosoft International, CA, USA). The sequences of the F and R primers used are given in Additional file 4. qRT-PCR was performed using a SYBR® Premix Ex TaqTM (Tli RNaseH Plus, Takara Bio, Inc, Otsu, Shiga, Japan) with the Step One Plus Real-Time System (Applied Biosystems, California, USA) in an Eco Real-Time PCR System (Illumina, USA ). The cycling conditions were 30 s at 95°C, followed by 40 cycles of 95°C for 5 s, 60°C for 15 s. Samples for qRT-PCR were run in 3 biological replicates with 3 technical replicates. Relative gene expression was calculated using ddCt algorithm. For the normalization of gene expression, citrus *actin* (GU911361.1) gene was used as an internal standard and the leaves from control plants were used as reference sample, which was set to 1.

### Experimental design and statistical analysis

There were 20 pots (40 seedlings) per treatment in a completely randomized design. Experiments were performed with 2–10 replicates. Results represented the mean ± SE. Statistical analyses of data were carried out by ANOVA tests. Means were separated by the least significant difference (LSD) test at *P* < 0.05 level.

## Competing interests

The authors declare that they have no competing interests.

## Authors’ contributions

CPZ carried out most of the experiments and drafted the manuscript. YPQ participated in the design of the study. XY participated in the sequence alignment. LTY participated in the design of the study and coordination. PG performed the statistical analysis. XXZ carried out the measurement of Mn and Chl. FJK carried out the cultivation of seedlings. LSC designed and directed the study and revised the manuscript. All authors have read and approved the final manuscript.

## Supplementary Material

Additional file 1 **Manganese (Mn)-toxicity symptoms on leaves of *Citrus grandis *and *C. sinensis*.** A: Control leaves of *C. grandis*; B: Mn-toxicity leaves of *C. grandis*; C: Control leaves of *C. sinensis*; D: Mn-toxicity leaves of *C. sinenis*. Click here for file

Additional file 2 **Homology of differentially expressed cDNA-AFLP fragments with known gene sequences in database using BLASTN algorithm along their expression patterns in Mn-toxicity leaves of *Citrus grandis*.** Click here for file

Additional file 3 **Homology of differentially expressed cDNA-AFLP fragments with known gene sequences in database using BLASTN algorithm along their expression patterns in Mn-toxicity leaves of *Citrus sinensis*.** Click here for file

Additional file 4 Specific primer pairs used for qRT-PCR expression analysis. Click here for file
