# Supplementary material for: Leaf cDNA-AFLP analysis of two citrus species differing in manganese tolerance in response to long-term manganese-toxicity
Source: BMC Genomics. 2013 Sep 14;14:621. doi: 10.1186/1471-2164-14-621 (PMC3847489; doi:10.1186/1471-2164-14-621)
Supplement: Additional file 1 — Manganese (Mn)-toxicity symptoms on leaves of Citrus grandis and C. sinensis. A: Control leaves of C. grandis; B: Mn-toxicity leaves of C. grandis; C: Control leaves of C. sinensis; D: Mn-toxicity leaves of C. sinenis. [file 1471-2164-14-621-S1.doc]

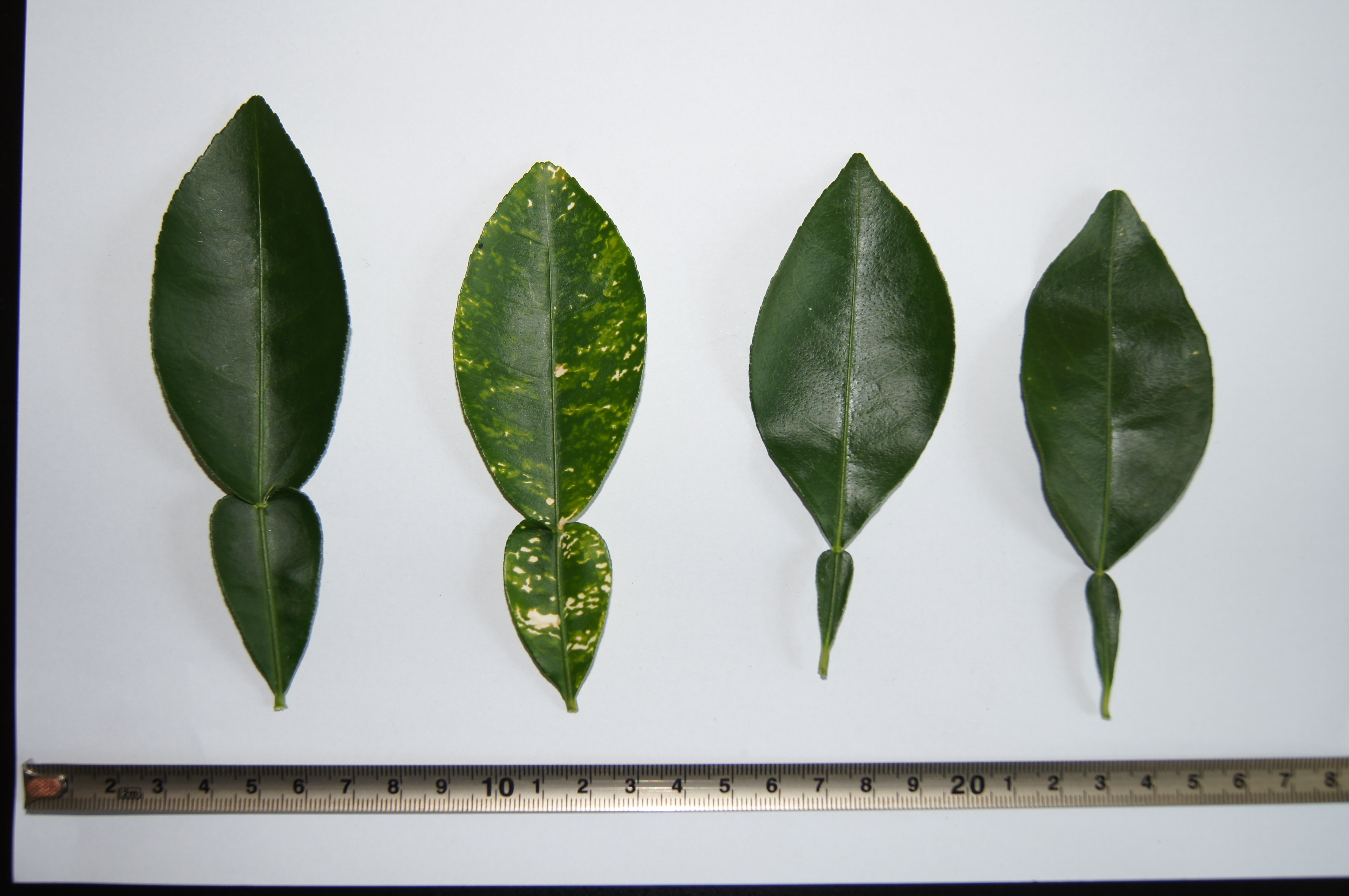


**A B C D**

**Additional file 1: Manganese (Mn)-toxicity symptoms on leaves of *Citrus grandis* and *C. sinensis*.** A: Control leaves of *C. grandis*; B: Mn-toxicity leaves of *C. grandis*; C: Control leaves of *Citrus sinensis*; D: Mn-toxicity leaves of *C. sinenis*.
