# Supplementary material for: Leaf cDNA-AFLP analysis of two citrus species differing in manganese tolerance in response to long-term manganese-toxicity
Source: BMC Genomics. 2013 Sep 14;14:621. doi: 10.1186/1471-2164-14-621 (PMC3847489; doi:10.1186/1471-2164-14-621)
Supplement: Additional file 2 — Homology of differentially expressed cDNA-AFLP fragments with known gene sequences in database using BLASTN algorithm along their expression patterns in Mn-toxicity leaves of Citrus grandis. [file 1471-2164-14-621-S2.docx]

**Additional file 2:**  **Homology of differentially expressed cDNA**-**AFLP fragments with known gene sequences in database using BLASTN algorithm along their expression patterns in Mn**-**toxicity leaves of** ***Citrus grandis***

| **TDF #** | **Size(bp)** | **Homology** | **Organism origin** | **E**-**value** | **Similarity(%)** | **Genebank ID** |
| --- | --- | --- | --- | --- | --- | --- |
| ***Biological regulation and signal transduction*** | | |  |  |  |  |
| 066-4 | 223 | Leucine-rich receptor-like protein kinase | *Arabidopsis thaliana* | 4E-15 | 60 | NP_177363.1 |
| 104-5 | 290 | Probable receptor-like protein kinase | *Cucumis sativus* | 2E-38 | 72 | XP_004159800.1 |
| 170-1 | 297 | Ser/Thr protein kinase isolog | *A. thaliana* | 4E-52 | 91 | BAD94000.1 |
| 199-1 | 274 | VH1-interacting kinase | *A. thaliana* | 5E-45 | 85 | NP_172853.1 |
| 165-2 | 171 | Calcium-dependent protein kinase 1-like | *C. sativus* | 5E-07 | 82 | XP_004162718.1 |
| 238-1 | 160 | OBP3-responsive gene 1 | *A. lyrata subsp.* | 3E-08 | 63 | XP_002864243.1 |
| **089**-**1** | **351** | **Mitogen**-**activated protein kinase 1** | ***A. lyrata subsp.*** | **4E**-**46** | **78** | **XP_002889793.1** |
| 044-1 | 284 | Protein phosphatase 2a, regulatory subunit, putative | *Ricinus communis* | 2E-08 | 59 | XP_002514974.1 |
| **09**-**2** | **187** | **Transcription factor jumonji domain**-**containing protein** | ***A. lyrata subsp.*** | **3E**-**8** | **48** | **XP_002892479.1** |
| **232**-**1** | **331** | **Myb family transcription factor** | ***A. thaliana*** | **5E**-**14** | **46** | **NP_187687.1** |
| 105-2 | 175 | Transcription factor ILR3 | *A. thaliana* | 5E-14 | 89 | NP_200279.1 |
| 156-2 | 289 | C3H4 type zinc finger protein | *A. thaliana* | 2E-19 | 49 | NP_189369.1 |
| 045-2 | 462 | Transcription factor, putative | *R. communis* | 8E-39 | 69 | XP_002528295.1 |
| 131-1 | 416 | DNA-binding storekeeper protein-related transcriptional regulator | *A. thaliana* | 2E-06 | 50 | NP_172618.1 |
| 100-2 | 601 | Calmodulin-binding transcription activator 5 | *A. thaliana* | 1E-39 | 69 | NP_193350.5 |
| 186-2 | 187 | Ca^2+^-binding protein | *Citrus sinensis* | 2E-28 | 98 | ABK06394.1 |
| **153**-**3** | **153** | **Calcium**-**binding EF**-**hand domain**-**containing protein** | ***A. thaliana*** | **5E**-**09** | **55** | **NP_564623.2** |
| 111-2 | 279 | Auxin-response factor | *C. sinensis* | 5E-51 | 99 | AEV43357.1 |
| **233**-**1** | **302** | **Auxin**-**induced protein 5NG4** | ***R. communis*** | **1E**-**53** | **93** | **XP_002517269.1** |
| 085-3 | 140 | Transducin/WD-40 repeat-containing protein | *A.thaliana* | 4E-09 | 67 | NP_563703.1 |
| 061-2 | 393 | VQ motif-containing protein | *A. thaliana* | 2E-08 | 75 | NP_565177.1 |
| 235-2 | 274 | Hsp70 nucleotide exchange factor fes1 | *Medicago truncatula* | 2E-43 | 86 | XP_003604937.1 |
| 099-2 | 222 | Phosphatidylinositol 4-kinase type 2-beta | *M. truncatula* | 2E-03 | 95 | XP_003610975.1 |
| ***Carbohydrate and energy metabolism*** | | |  |  |  |  |
| **065**-**1** | **245** | **ATP synthase subunit alpha (Atp 1)** | ***Citrus limon*** | **3E**-**41** | **95** | **ADL63180.1** |
| **01**-**1** | **336** | **ATP synthase subunit alpha** | ***M. truncatula*** | **5E**-**31** | **81** | **XP_003588326.1** |
| **116**-**1** | **324** | **Aconitate hydratase 2** | ***Citrus clementina*** | **4E**-**44** | **94** | **CBE71058.1** |
| **178**-**1** | **146** | **NADPH**-**ferrihemoprotein reductase** | ***A. thaliana*** | **3E**-**10** | **71** | **NP_194750.1** |
| **236**-**1** | **199** | **Cytochrome c biogenesis orf256** | ***A. thaliana*** | **9E**-**09** | **97** | **NP_085546.2** |
| 244-1 | 418 | 2-phospho-D-glycerate hydrolase | *Citrus trifoliata* | 6E-60 | 92 | ADD12953.1 |
| 197-1 | 193 | Cytochrome P450 | *R. communis* | 6E-25 | 81 | XP_002540268.1 |
| 058-2 | 209 | Thioredoxin *m* | *Populus trichocarpa* | 7E-12 | 78 | XP_002329095.1 |
| 085-2 | 124 | Thioredoxin *m*4, chloroplastic-like | *Glycine max* | 1E-15 | 76 | XP_003541848.1 |
| 013-3 | 312 | Trehalose-6-phosphate synthase | *Ginkgo biloba* | 1E-40 | 72 | AAX16014.1 |
| 168-1 | 346 | ATP sulfurylase 1 | *A. thaliana* | 2E-58 | 87 | NP_188929.1 |
| 185-4 | 238 | UDP-glycosyltransferase 76F1-like | *G. max* | 5E-27 | 72 | XP_003537476.1 |
| 121-1 | 303 | Carboxypeptidase regulatory region-containing protein, putative | *R. communis* | 1E-04 | 88 | XP_002515261.1 |
| ***Nucleic acid metabolism*** | | |  |  |  |  |
| 134-2 | 243 | THO complex, subunit 5 | *A. thaliana* | 2E-11 | 47 | NP_568616.1 |
| 139-1 | 190 | DNA polymerase phi subunit | *A. thaliana* | 3E-05 | 39 | NP_201247.1 |
| 165-1 | 306 | Histone H4 | *Zea mays* | 1E-54 | 98 | ACG30677.1 |
| 200-1 | 275 | DNA (cytosine-5)- methyltransferase DRM2-like | *Vitis vinifera* | 2E-37 | 80 | XP_002264226.1 |
| 200-2 | 226 | Luc7-like protein 3-like | *V. vinifera* | 3E-23 | 91 | XP_002267755.1 |
| ***Protein metabolism*** | | |  |  |  |  |
| 06-1 | 240 | Ribosomal protein S3 | *C. sinensis* | 2E-32 | 92 | YP_740514.1 |
| 097-1 | 223 | Ribosomal protein S8 | *C. sinensis* | 1E-17 | 100 | YP_740511.1 |
| 134-1 | 291 | 60S ribosomal protein L2, mitochondrial-like | *V. vinifera* | 3E-09 | 79 | XP_002279271.1 |
| **140**-**1** | **267** | **30S ribosomal protein S13** | ***Camellia sinensis*** | **5E**-**14** | **94** | **AEC10995.1** |
| 011-2 | 267 | Similar to translation initiation factor IF2 | *A. thaliana* | 0.014 | 49 | AAD50011.1 |
| 181-2 | 251 | Translation elongation factor-1 alpha, partial | *Citrus maxima* | 1E-44 | 97 | AFB18314.1 |
| 090-1 | 246 | Tetratricopeptide repeat-containing protein | *A. thaliana* | 3E-15 | 75 | NP_199947.1 |
| **245**-**1** | **311** | **ATP**-**dependent Clp protease** | ***Eriobotrya japonica*** | **4E**-**35** | **85** | **ACX50407.1** |
| **044**-**2** | **174** | **Cysteine proteinase** | ***Lotus japonicus*** | **1E**-**12** | **74** | **BAF56427.1** |
| **098**-**2** | **202** | **Xylem cysteine proteinase 2** | ***Z. mays*** | **4E**-**18** | **67** | **AFW74997.1** |
| 104-6 | 220 | Carboxyl-terminal peptidase | *A. thaliana* | 4E-35 | 95 | AAM65243.1 |
| 107-1 | 252 | Papain family cysteine protease | *A. thaliana* | 3E-28 | 83 | NP_567489.1 |
| 233-3 | 126 | Cathepsin B-like cysteine proteinase like protein | *A. thaliana* | 1E-11 | 91 | BAD94873.1 |
| 221-1 | 205 | Alpha/beta-hydrolase-like protein | *A. thaliana* | 4E-24 | 79 | NP_178144.1 |
| **148**-**1** | **334** | **MND1**-**interacting protein 1** | ***A. thaliana*** | **2E**-**27** | **58** | **NP_174531.1** |
| **240**-**1** | **320** | **Ubiquitin**-**conjugating enzyme E2 10** | ***A. thaliana*** | **5E**-**60** | **99** | **NP_568788.1** |
| 03-2 | 376 | Ubiquitin-protein ligase | *R. communis* | 5E-23 | 64 | XP_002518834.1 |
| 138-2 | 219 | BTB and MATH domain-containing protein | *R. communis* | 5E-12 | 76 | XP_002525692.1 |
| 247-1 | 454 | Chorismate synthase | *A. thaliana* | 7E-61 | 86 | NP_564534.1 |
| 216-3 | 382 | Cystathionine β-synthase domain-containing protein | *A. thaliana* | 8E-23 | 61 | NP_194476.2 |
| 061-3 | 208 | 2-oxoglutarate (2OG) and Fe(II)-dependent oxygenase-like protein | *A. thaliana* | 5E-06 | 70 | NP_566623.1 |
| ***Lipid metabolism*** | | |  |  |  |  |
| **153**-**2** | **296** | **Lecithin**-**cholesterol acyltransferase**-**like 1** | ***A. thaliana*** | **3E**-**29** | **63** | **NP_564286.1** |
| ***Cell wall metabolism*** | | |  |  |  |  |
| 037-3 | 241 | Alpha-1,2-fucosyltransferase | *Populus tremula × Populus alba* | 1E-26 | 74 | ABS70459.1 |
| 080-1 | 163 | Caffeic acid O-methyltransferase | *C. sinensis x Citrus reticulata* | 1E-05 | 53 | ABP94018.1 |
| 069-8 | 254 | O-fucosyltransferase family protein | *A. thaliana* | 4E-37 | 86 | NP_201350.2 |
| 044-4 | 199 | Cellulose synthase-like protein | *Populus tomentosa* | 5E-17 | 62 | AFZ78580.1 |
| 05-1 | 253 | Protein SAH7 | *A. thaliana* | 3E-11 | 76 | NP_567338.1 |
| 151-1 | 152 | 4-coumarate-CoA ligase 3 | *A. thaliana* | 2E-13 | 74 | NP_849844.1 |
| **043**-**1** | **434** | **Glycoside hydrolase family 28 protein** | ***A. lyrata subsp.*** | **1E**-**73** | **85** | **XP_002876618.1** |
| **242**-**1** | **401** | **Cell wall**-**associated hydrolase** | ***M. truncatula*** | **2E**-**49** | **79** | **XP_003637074.1** |
| **158**-**3** | **178** | **Protein trichome birefringence**-**like 39** | ***A. thaliana*** | **6E**-**08** | **48** | **NP_565975.1** |
| 04-4 | 165 | Glucan endo-1,3-beta-glucosidase precursor, putative | *R. communis* | 5E-21 | 76 | XP_002518930.1 |
| ***Stress responses*** | | |  |  |  |  |
| **103**-**2** | **237** | **Catalase** | ***C. maxima*** | **2E**-**41** | **94** | **ACY30463.1** |
| **160**-**1** | **318** | **Nudix hydrolase 19** | ***A. thaliana*** | **3E**-**50** | **78** | **NP_197507.1** |
| **164**-**4** | **333** | **Putative senescence**-**associated protein** | ***Pyrus communis*** | **1E**-**50** | **99** | **AAR25995.1** |
| 098-1 | 253 | Monodehydroascorbate reductase | *A. thaliana* | 2E-41 | 89 | BAA12349.2 |
| 104-1 | 276 | Peroxidase 42 | *A. thaliana* | 3E-44 | 82 | NP_567641.1 |
| 227-1 | 365 | Glutathione S-transferase Tau2 | *C. sinensis* | 7E-54 | 91 | ACI42271.1 |
| 130-2 | 250 | NADP-dependent alkenal double bond reductase P2 | *V. vinifera* | 9E-25 | 67 | XP_002279529.1 |
| **054**-**1** | **234** | **ALG2**-**interacting protein X** | ***Nicotiana tabacum*** | **1E**-**30** | **86** | **BAD15108.1** |
| 158-2 | 314 | Heat shock protein-related | *A. thaliana* | 4E-22 | 82 | CAM31939.1 |
| 160-5 | 205 | Heat shock protein 60-3A | *A. thaliana* | 2E-23 | 81 | NP_566466.1 |
| 237-6 | 142 | Heat shock protein 70 | *Nicotiana benthamiana* | 5E-06 | 78 | BAD02271.1 |
| 156-1 | 382 | Stromal 70 kDa heat shock-related protein, chloroplastic-like | *V. vinifera* | 2E-71 | 96 | XP_002279101.1 |
| 198-1 | 140 | Phosphoethanolamine/phosphocholine phosphatase, putative | *R. communis* | 7E-16 | 89 | XP_002527425.1 |
| ***Cell transport*** | | |  |  |  |  |
| **073**-**1** | **282** | **Citrus sucrose transporter 1** | ***C. sinensis*** | **1E**-**42** | **100** | **AAM29150.1** |
| 208-1 | 313 | ABC-transporter-like protein | *A. thaliana* | 2E-25 | 83 | XP_003620472.1 |
| 248-1 | 274 | ABC transporter family protein | *A. lyrata subsp.* | 4E-14 | 52 | XP_002873263.1 |
| 234-2 | 204 | Magnesium transporter CorA-like protein | *A. thaliana* | 4E-27 | 80 | NP_178511.2 |
| 216-2 | 239 | Cyclic nucleotide gated channel 9 | *A. thaliana* | 2E-10 | 46 | NP_001190873.1 |
| 242-2 | 229 | Protease inhibitor/seed storage/lipid transfer protein (LTP) family protein | *A. thaliana* | 2E-13 | 55 | NP_565872.1 |
| 222-2 | 236 | Exocyst complex component 7-like | *G. max* | 3E-19 | 62 | XP_003551759.1 |
| 160-6 | 365 | Regulator of Vps4 activity in the MVB pathway protein | *A. thaliana* | 3E-57 | 78 | NP_564235.1 |
| ***Other and unknown biological processes*** | | |  |  |  |  |
| **09**-**1** | **313** | **IFA binding protein** | ***Lilium longiflorum*** | **9E**-**11** | **50** | **ABM68547.1** |
| **157**-**1** | **248** | **Mitochondrial protein, putative** | ***M. truncatula*** | **2E**-**19** | **98** | **XP_003588355.1** |
| 118-1 | 304 | LisH/CRA/RING-U-box domain-containing protein | *A. thaliana* | 8E-22 | 49 | NP_196525.1 |
| 063-1 | 201 | Anthranilate phosphoribosyltransferase-like protein | *A. thaliana* | 3E-24 | 77 | NP_196801.1 |
| 133-3 | 445 | Haloacid dehalogenase-like hydrolase domain-containing protein | *A. thaliana* | 4E-69 | 75 | NP_850072.1 |
| **118**-**3** | **146** | **Uncharacterized protein LOC100795351** | ***G. max*** | **2E**-**13** | **82** | **XP_003541217.1** |
| 027-1 | 268 | Predicted protein | *P. trichocarpa* | 4E-25 | 63 | XP_002330640.1 |
| **028**-**1** | **234** | **Hypothetical protein MTR_1g005050** | ***M. truncatula*** | **2E**-**20** | **94** | **XP_003588264.1** |
| 04-3 | 266 | Uncharacterized proteinAt5g57460 | *A. thaliana* | 2E-14 | 57 | NP_200555.1 |
| 055-1 | 339 | Uncharacterized protein LOC100259944 | *V. vinifera* | 1E-22 | 54 | XP_002272156.1 |
| 057-2 | 325 | Unnamed protein product LOC100261548 | *V. vinifera* | 1E-14 | 48 | CBI39111.3 |
| **069**-**4** | **291** | **Hypothetical protein SORBIDRAFT_0070s002020** | ***Sorghum bicolor*** | **4E**-**40** | **81** | **XP_002489102.1** |
| **092**-**1** | **112** | **Uncharacterized protein LOC101218508, partial** | ***C. sativus*** | **3E**-**11** | **90** | **XP_004154316.1** |
| **100**-**3** | **163** | **Hypothetical protein MTR_5g051010** | ***M. truncatula*** | **8E**-**18** | **94** | **XP_003614384.1** |
| **109**-**3** | **179** | **Unknown protein** | ***G. max*** | **3E**-**15** | **85** | **ACU14517.1** |
| **111**-**1** | **297** | **Hypothetical protein MTR_5g051140** | ***M. truncatula*** | **3E**-**14** | **93** | **XP_003614395.1** |
| **117**-**1** | **317** | **Uncharacterized protein AT5G48470** | ***A. thaliana*** | **5E**-**29** | **87** | **NP_568697.1** |
| **138**-**1** | **258** | **Hypothetical protein CHLPROCp009, partial** | ***Auxenochlorella protothecoides*** | **1E**-**22** | **67** | **AGB85039.1** |
| 139-2 | 173 | Uncharacterized protein AT4G09830 | *A. thaliana* | 8E-23 | 82 | NP_192721.1 |
| 163-2 | 287 | Hypothetical protein | *A. thaliana* | 7E-43 | 84 | CAB10396.1 |
| **163**-**3** | **310** | **Uncharacterized protein LOC100810244** | ***G. max*** | **4E**-**39** | **85** | **XP_003541241.1** |
| 181-1 | 317 | Conserved hypothetical protein | *R. communis* | 8E-32 | 88 | XP_002527256.1 |
| **188**-**1** | **253** | **Hypothetical protein ARALYDRAFT_475506** | ***A. lyrata subsp.*** | **2E**-**21** | **73** | **XP_002888301.1** |
| **214**-**1** | **432** | **Hypothetical protein** | ***A. thaliana*** | **1E**-**83** | **92** | **BAF01964.1** |
| **229**-**1** | **201** | **Hypothetical protein MTR_5g051120** | ***M. truncatula*** | **1E**-**23** | **89** | **XP_003614393.1** |
| **237**-**5** | **201** | **Hypothetical protein SORBIDRAFT_1292s002010** | ***S. bicolor*** | **2E**-**21** | **91** | **XP_002488951.1** |
| 085-1 | 216 | Hypothetical protein PRUPE_ppa013598mg | *Prunus persica* | 2E-14 | 73 | EMJ24707.1 |
| **134**-**3** | **205** | **Unknown** | ***M. truncatula*** | **5E**-**10** | **93** | **AFK40983.1** |

TDF: Transcript-derived fragment; **Bold and blue: Up**-**regulated TDFs;** Black: Down-regulated TDFs.
