# Supplementary material for: Leaf cDNA-AFLP analysis of two citrus species differing in manganese tolerance in response to long-term manganese-toxicity
Source: BMC Genomics. 2013 Sep 14;14:621. doi: 10.1186/1471-2164-14-621 (PMC3847489; doi:10.1186/1471-2164-14-621)
Supplement: Additional file 3 — Homology of differentially expressed cDNA-AFLP fragments with known gene sequences in database using BLASTN algorithm along their expression patterns in Mn-toxicity leaves of Citrus sinensis. [file 1471-2164-14-621-S3.docx]

**Additional file 3:**  **Homology of differentially expressed cDNA**-**AFLP fragments with known gene sequences in database using BLASTN algorithm along their expression patterns in Mn**-**toxicity leaves of** ***Citrus sinensis***

| **TDF #** | **Size(bp)** | **Homology** | **Organism origin** | **E**-**value** | **Similarity(%)** | **Genebank ID** |
| --- | --- | --- | --- | --- | --- | --- |
| ***Biological regulation and signal transduction*** | | |  |  |  |  |
| **199a** | **274** | **VH1**-**interacting kinase** | ***Arabidopsis thaliana*** | **5E**-**45** | **85** | **NP_172853.1** |
| **100b** | **601** | **Calmodulin**-**binding transcription activator 5** | ***A. thaliana*** | **1E**-**39** | **69** | **NP_193350.5** |
| **105b** | **175** | **Transcription factor ILR3** | ***A. thaliana*** | **5E**-**14** | **89** | **NP_200279.1** |
| 131a | 416 | DNA-binding storekeeper protein-related transcriptional regulator | *A. thaliana* | 2E-06 | 50 | NP_172618.1 |
| 233a | 302 | Auxin-induced protein 5NG4 | *Ricinus communis* | 1E-53 | 93 | XP_002517269.1 |
| ***Carbohydrate and energy metabolism*** | | |  |  |  |  |
| **065a** | **245** | **Atp1** | ***Citrus limon*** | **3E**-**41** | **95** | **ADL63180.1** |
| **029a** | **256** | **Cytochrome P450, family 96, subfamily A, polypeptide 9** | ***A. thaliana*** | **1E**-**31** | **71** | **NP_195658.3** |
| ***Nucleic acid metabolism*** | | |  |  |  |  |
| 134b | 243 | THO complex, subunit 5 | *A. thaliana* | 2E-11 | 47 | NP_568616.1 |
| 165a | 306 | Histone H4 | *Zea mays* | 1E-54 | 98 | ACG30677.1 |
| ***Protein metabolism*** | | |  |  |  |  |
| 134a | 291 | 60S ribosomal protein L2, mitochondrial-like | *Vitis vinifera* | 3E-09 | 79 | XP_002279271.1 |
| ***Cell transport*** | | |  |  |  |  |
| 234b | 204 | Magnesium transporter CorA-like-like protein | *A. thaliana* | 4E-27 | 80 | NP_178511.2 |
| ***Other and unknown biological processes*** | | |  |  |  |  |
| **134c** | **205** | **Mitochondrial protein, putative** | ***Medicago truncatula*** | **4E**-**08** | **91** | **XP_003588355.1** |
| **136a** | **197** | **Hypothetical protein VITISV_015730** | ***V. vinifera*** | **1E**-**04** | **85** | **CAN70065.1** |
| 181a | 317 | Conserved hypothetical protein | *R. communis* | 8E-32 | 88 | XP_002527256.1 |
| 056b | 234 | Hypothetical protein MTR_4g091430 | *M. truncatula* | 1E-39 | 94 | XP_003608262.1 |

TDF: Transcript-derived fragment; **Bold and blue: Up**-**regulated TDFs;** Black: Down-regulated TDFs.
