# Supplementary material for: Leaf cDNA-AFLP analysis of two citrus species differing in manganese tolerance in response to long-term manganese-toxicity
Source: BMC Genomics. 2013 Sep 14;14:621. doi: 10.1186/1471-2164-14-621 (PMC3847489; doi:10.1186/1471-2164-14-621)
Supplement: Additional file 4 — Specific primer pairs used for qRT-PCR expression analysis. [file 1471-2164-14-621-S4.doc]

**Additional file 4: Specific primer pairs used for qRT-PCR expression analysis**

| ***TDF # or gene*** | *Forward primers (5´→3´)* | *Reverse primers (5´→3´)* |
| --- | --- | --- |
| 01-1 | CGAAGGAACGCCAAAAGC | CGTAGACCAAGGGCGAACA |
| 043-1 | TGGAATGCCCACAAAACAAC | ATACCCTCCTCTGCCTACGG |
| 063-1 | TATTGAGCTGGCGGGATC | AAGAGCAAGCACCTGGAAA |
| 065-1 | AGTCCTGAGTAAACCAAAAG | GTGGCTGCTACAAGAATG |
| 098-1 | GAAGCCCCAGGAAAGTGTG | TCCCGCTTTCTACAAGGAC |
| 098-2 | CGCAGCAGTTCTTCCATACCAA | AGAAGCCAACCCTAGTACGAGA |
| 103-2 | ACTGCGATCCAATTCCTCA | CCTTCCATGTGCCTGTAATCT |
| 104-1 | CCCAAGGCTGTTCAGTATGTG | TTGTCCTCTTGTCGGTGGC |
| 130-2 | GGTTTCTTGCCGGTGATT | AGGGCTGATGGAGCTTTCT |
| 139-1 | GTGATGAGGACGAACAGGC | CGAAGAAGCACTTGGAGCAG |
| 160-6 | TTCTCGCATTTCTGTTGGAC | TTCGGGTGGAGCATGTTATA |
| 227-1 | TCATCCACAACGGCAGACC | TCGCCTCCTCCGAAGAAA |
| 232-1 | GGCACTCCACCAACAGCAGA | TAGCGGGCGACCCAAAAT |
| 242-1 | GGGCGTTAGAGCATTGAGA | CTTGGCTACCCAGCGTTTA |
| 248-1 | GGCGAGAACAAAATCAGACC | ATCCGACGGCTAGACTTCC |
| 065a | AGTCCTGAGTAAACCAAAAG | GTGGCTGCTACAAGAATG |
| *Actin* | AGAACTATGAACTGCCTGATGGC | GCTTGGAGCAAGTGCTGTGATT |

TDFs: Transcript-derived fragments; All these primers were designed based on the results of identified changed genes in *Citrus grandis* and showed no relative to the genes changed in *C. sinensis* except for 065-1 (*C. grandis*) and 065a (*C. sinensis*).
